# Xanthan Gum/Lignin Double-Interpenetrating Network Cryogels: Preparation, Characterization, and Water Purification Performance

**DOI:** 10.3390/gels12100915

**Published:** 2026-10-11

**Authors:** Maria Marinela Lazar, Doina Humelnicu, Irina Elena Raschip, Raluca-Elena Avram, Maria Valentina Dinu

**Affiliations:** 1 Department of Functional Polymers, “Petru Poni” Institute of Macromolecular Chemistry, Grigore Ghica Voda Alley 41A, 700487 Iasi, Romania; iecoj@icmpp.ro (I.E.R.); munteanu.raluca@icmpp.ro (R.-E.A.); vdinu@icmpp.ro (M.V.D.); 2 Faculty of Chemistry, Alexandru Ioan Cuza University of Iasi, Carol I Bd. 11, 700506 Iasi, Romania; doinah@uaic.ro

**Keywords:** xanthan gum, lignin, interpenetrating polymer networks, cryogels, Cu(II), SO_4_^2−^, antibacterial activity

## Abstract

The development of multifunctional, bio-based materials capable of simultaneously addressing microbial contamination and inorganic pollutants is of increasing interest for sustainable water purification. In this study, xanthan gum (XG)/lignin(LN) double-interpenetrating network (DN) cryogels were prepared and investigated as porous, multifunctional materials for antimicrobial applications and water purification. The cryogel architecture combines the abundant oxygen-containing functional groups of XG and LN with an interconnected porous network generated under cryogenic conditions. The prepared networks were comprehensively characterized using Fourier-transform infrared spectroscopy (FTIR), scanning electron microscopy (SEM), energy-dispersive X-ray spectroscopy (EDX), dynamic vapor sorption (DVS), swelling analysis, and uniaxial mechanical testing to evaluate their structural, morphological, physicochemical, and mechanical properties. Their Cu^2+^ sorption performance was subsequently assessed by atomic absorption spectroscopy (AAS), and the experimental equilibrium data were analyzed using the Langmuir, Freundlich, and Sips isotherm models. The single-network (SN) XG-based materials exhibited Cu^2+^ sorption capacities of 62.13–109.81 mg/g, whereas the DN cryogels showed higher capacities of 111.87–120.84 mg/g. The Sips model provided the best description of the equilibrium data, with (R^2^) values of 0.991–0.994 and substantially lower (χ^2^) values than the Langmuir and Freundlich models, indicating heterogeneous sorption sites with finite saturation capacity. Importantly, the Cu^2+^-loaded SN and DNs exhibited sorption efficiency toward sulfate ions, demonstrating their potential for sequential contaminant removal. Sulfate sorption capacities reached 694.53 mg/g for the SN.X2/Cu^2+^ composite and up to 812.53 mg/g for the DN.XL3/Cu^2+^ composite. In addition, the prepared materials demonstrated antimicrobial activity against two Gram-negative (*Escherichia coli*, *Salmonella Typhimurium*) and two Gram-positive (*Staphylococcus aureus*, *Listeria monocytogenes*) bacterial strains. The results demonstrate that XG/LN DN cryogels combine effective metal-ion and sulfate sorption with antimicrobial activity, providing promising bio-based platforms for water purification and potentially extending the functional lifetime of the material through sequential contaminant removal.

## 1. Introduction

Access to safe drinking water remains a major global challenge, particularly in regions affected by industrial activities, environmental accidents, and natural disasters. Water contamination by heavy metal ions (HMIs), inorganic ions, and pathogenic microorganisms poses serious risks to both human health and ecosystems. Among these pollutants, HMIs are of particular concern because of their persistence, non-biodegradability, bioaccumulation potential, and toxicity even at low concentrations [1,2]. Recent studies have highlighted the increasing prevalence of HMIs contamination in aquatic environments, including Romanian water resources, where elevated concentrations of chromium, iron, zinc, cobalt, and copper have been reported [3,4].

Copper is an essential trace element involved in numerous physiological processes, including enzymatic activity, cellular metabolism, and hormone regulation. However, excessive copper exposure can cause severe environmental and health problems, affecting growth, reproduction, neurological functions, and metabolic processes in living organisms [2]. In humans, elevated copper levels have been associated with dyslipidemia, liver disorders, and Wilson’s disease [2,5]. Consequently, the development of efficient and sustainable technologies for copper removal from contaminated waters remains an important research priority.

Various treatment methods have been employed for the removal of HMIs from wastewater, including chemical precipitation, membrane filtration, coagulation–flocculation, ion exchange, electrochemical treatment, and adsorption [6,7,8,9]. Among these approaches, adsorption is generally considered one of the most attractive techniques due to its simplicity, high efficiency, operational flexibility, and relatively low cost [7,8,9,10]. The effectiveness of adsorption processes largely depends on the physicochemical characteristics of the adsorbent, particularly the accessibility of functional groups, porosity, mechanical stability, and regeneration capability [6,7,8,9,10].

In recent years, hydrogels and cryogels have emerged as promising adsorbent materials for water purification applications [11,12,13,14,15,16,17,18,19]. Cryogels are macroporous polymeric networks formed through cryogelation, a process in which gelation occurs in partially frozen systems and ice crystals act as porogens [15,18,20,21]. Their interconnected porous structure facilitates rapid mass transfer, high water permeability, and efficient interaction between pollutants and adsorption sites [10,15,18,22]. Compared with conventional hydrogels, cryogels exhibit superior permeability, elasticity, shape-memory properties, and resistance to mechanical deformation, making them particularly suitable for dynamic water treatment systems [10,15,18,20,21,22].

The growing demand for environmentally friendly materials has stimulated the development of adsorbents based on renewable biopolymers. Natural polymers are attractive because they are biodegradable, biocompatible, non-toxic, and readily available from sustainable sources [23,24]. Among them, polysaccharides have received considerable attention due to their abundance of functional groups, including hydroxyl, carboxyl, amino, and sulfate groups, which can effectively interact with HMIs through electrostatic attraction, complexation, and ion-exchange mechanisms [14,23,25,26].

Xanthan gum (XG), a microbial extracellular polysaccharide produced by *Xanthomonas campestris*, possesses remarkable rheological properties, high viscosity, excellent stability over broad pH and temperature ranges, and a large number of reactive functional groups [27,28,29]. These characteristics make XG a valuable platform for the fabrication of adsorbent materials. Several XG-based hydrogels have demonstrated high adsorption capacities and good reusability for the removal of HMIs from aqueous solutions [30,31].

Lignin (LN) represents another attractive renewable resource for environmental applications. As the second most abundant natural polymer after cellulose, LN is a complex 3D polyphenolic biopolymer characterized by antioxidant, antimicrobial, and stabilizing properties [16,24,28,32]. The presence of phenolic and aliphatic hydroxyl groups, methoxy groups, and aromatic structures provides multiple binding sites for pollutant adsorption [33]. Nevertheless, LN alone often exhibits limited adsorption efficiency and regeneration performance, which can be improved through chemical modification or incorporation into composite polymeric networks [34].

An effective approach for enhancing the mechanical stability and adsorption performance of hydrogel-based materials is the development of interpenetrating polymer networks (IPNs). IPNs are composed of two or more polymer networks that are physically interconnected at the molecular level without the formation of covalent bonds between the individual networks, thereby providing enhanced structural integrity and functional properties [35]. In particular, double-interpenetrating network (DN) hydrogels and cryogels have been reported to exhibit superior mechanical strength, deformation resistance, and adsorption capacity compared with their corresponding SN counterparts [36,37]. These architectures are particularly promising for water treatment applications, as they can simultaneously provide high porosity, structural robustness, and the potential for regeneration and reuse.

Although numerous studies have investigated the development of polysaccharide-based cryogels, studies addressing DN cryogels based on XG and LN remain scarce. The combination of the hydrophilic and ion-binding properties of XG with the polyphenolic structure and functional groups of LN is expected to induce synergistic effects, leading to enhanced adsorption performance and improved mechanical stability.

The aim of this study was to develop novel DN cryogels comprising XG as the first polymer network and LN as the second network, with epichlorohydrin (ECH) employed as the cross-linking agent. ECH is a versatile and widely used cross-linking agent for natural polymers, particularly polysaccharides such as cellulose, chitosan, dextran, pectin, and alginate, enabling the preparation of microspheres, hydrogels, membranes, films, and porous materials with enhanced water resistance and structural stability [38,39,40,41,42,43,44]. Its effectiveness is particularly relevant in alkaline media, where deprotonated hydroxyl groups of polysaccharides can react with the epoxide functionality of ECH, promoting covalent cross-linking and the formation of three-dimensional polymer networks. These ECH-cross-linked biomaterials have been investigated for diverse applications, including drug immobilization and controlled delivery, tissue engineering scaffolds, adsorption of various pollutants, agricultural applications, food processing, antimicrobial packaging, and oil–water separation [38,39,40,41,42,43,44,45,46,47]. Nevertheless, the use of ECH requires careful consideration of residual cross-linker because unreacted ECH is associated with toxicological concerns, including cytotoxicity and irritation, and has been described as a potential human carcinogen [48]. Thus, the safety of ECH-cross-linked biopolymer materials depends critically on achieving sufficiently complete reaction and implementing appropriate post-cross-linking washing and purification procedures to remove unreacted ECH. Importantly, several studies indicate that when ECH is effectively consumed during cross-linking, the resulting polymeric networks exhibited biocompatibility; for example, ECH-cross-linked polyglycerol hydrogels have demonstrated cytocompatibility [49], while long-term clinical evaluation of ECH-modified porcine aortic valve implants showed excellent performance and durability over 15 years [50]. In the present work, ECH was selected because it enables efficient formation of a covalently cross-linked XG network at subzero temperatures. The resulting cryogels were comprehensively characterized after the purification step using FTIR, SEM, EDX, DVS, swelling analysis, and mechanical compression testing. Their adsorption performance toward Cu^2+^ ions was assessed as a function of the sorbent dose, contact time, initial metal-ion concentration, the presence of competing ions, and reusability over multiple sorption–desorption cycles. Furthermore, the potential application of Cu^2+^-loaded SN and DN cryogels in a sequential contaminant removal process was investigated. The antimicrobial activity of the developed materials was also evaluated against *Escherichia coli*, *Salmonella Typhimurium*, *Staphylococcus aureus*, and *Listeria monocytogenes* bacterial strains.

## 2. Results and Discussion

### 2.1. Preparation of SN and DN Cryogels

**Figure 1:**
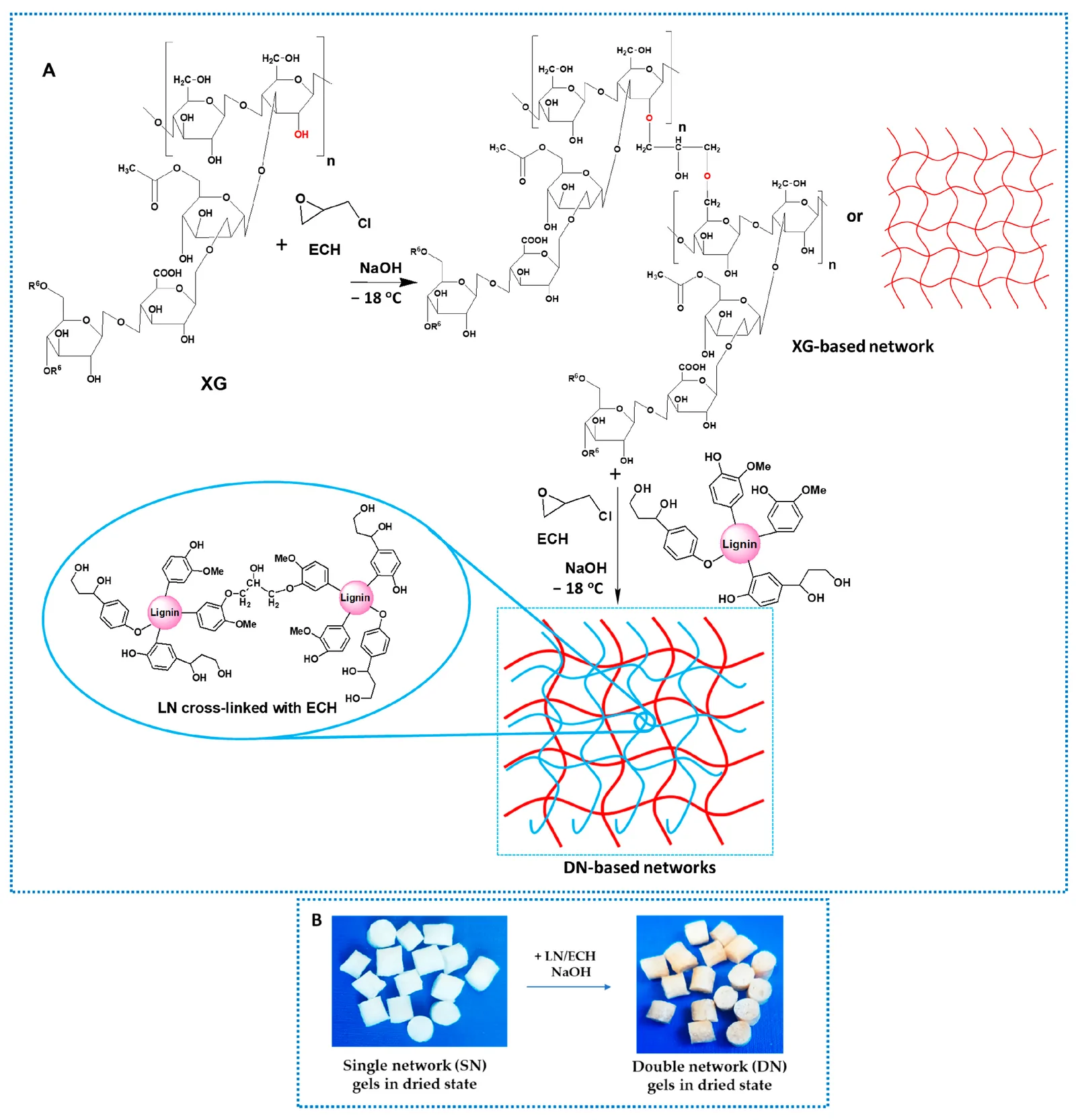
Schematic representation of the sequential formation of XG/LN double-network (DN) cryogels: (**A**) formation of the first XG-based network by ECH-mediated covalent cross-linking of XG through cryogelation at −18 °C, followed by swelling of the preformed XG network with an alkaline solution containing LN and ECH, and a second cryogelation step at −18 °C, during which LN is cross-linked in situ with ECH through its available hydroxyl functionalities, resulting in the formation of the second LN-based network within the pre-existing XG network; (**B**) optical images of the XG-based SN cryogel in the dried state before and after incorporation of the LN-based second network, indicating the characteristic change in appearance from white to light brown following LN incorporation.

The proposed sequential strategy for the preparation of DN cryogels, schematically presented in Figure 1A, involved the formation of the first XG-based network by cross-linking XG with ECH through cryogelation at −18 °C, followed by the generation of the second LN-based network within the preformed XG network. Specifically, an alkaline solution containing LN and ECH was used to swell the preformed XG network, after which a second cryogelation step at −18 °C was performed to generate the LN-based network in situ within the pre-existing XG network, thus yielding the interpenetrating DN structure. The optical images of the XG-based SN cryogel in the dried state, obtained before and after incorporation of the LN-based second network (Figure 1B), provide visual evidence of the formation of the XG/LN dual-network architecture. In particular, the transition in color from white to light brown following the incorporation of LN indicates the successful integration of the LN network into the preformed XG matrix.

The preparation of the SN and DN cryogels was designed to establish a relationship between the cross-linking conditions, network architecture, and structural stability of the resulting materials. The first objective was to investigate the influence of the ECH content, used as the covalent cross-linking agent, on the formation and integrity of the XG network and, subsequently, to determine how the incorporation of LN as a second network affects the gel fraction yield (GFY) and incorporation efficiency of the LN component. For the SN systems, the ECH ratio was varied over a broad range (Table 1), while maintaining constant XG concentration. Based on the optimized SN formulation, a second network containing LN was subsequently introduced to obtain the DN cryogels. In these systems, the ECH:OH molar ratio was varied from 8:100 to 20:100 to evaluate the efficiency of LN cross-linking and its retention within the preformed XG network.

For the SN cryogels, the XG concentration was maintained at 6 wt.%, while the ECH:XG molar ratio was varied from 3:100 to 30:100 (Table 1). An overall increase in GFY was observed with increasing ECH content. The GFY remained relatively constant at approximately 80% for ECH ratios between 3:100 and 10:100 but increased markedly to 89.16 ± 0.51% and 91.24 ± 1.06% at ratios of 15:100 and 20:100, respectively. Further increasing the ECH ratio to 30:100 resulted in a slightly lower GFY of 88.92 ± 3.08%, indicating that the increase in GFY was not directly proportional to the cross-linker concentration at the highest ECH level. The obtained GFY values ranged from approximately 79 to 91%, but no clear systematic trend between ECH concentration and GFY was observed. In particular, the GFY values obtained at ECH:XG ratios of 3:100, 6:100, and 10:100 were comparable, within the experimental variability, with values of approximately 80%. Although higher GFY values were obtained at ECH:XG ratios of 15:100 and 20:100 (89.16 ± 0.51% and 91.24 ± 1.06%, respectively), the relatively large variability observed for some formulations and the absence of a consistent trend indicate that GFY alone cannot be directly correlated with the ECH concentration. At the highest ECH:XG ratio of 30:100, the GFY was 88.92 ± 3.08%, further supporting the absence of a proportional relationship between cross-linker concentration and network yield. However, despite the relatively high GFY obtained at ECH ratios of 20:100 and 30:100, the resulting cryogels exhibited poor mechanical integrity and were fragile and prone to disintegration. This observation indicates that GFY alone does not necessarily reflect the structural stability of the resulting cryogel network. Therefore, the selection of the formulation for subsequent DN preparation was not based solely on GFY, but rather on the combined consideration of network yield, physical integrity, and suitability of the porous XG network for incorporation of the second network. On this basis, the ECH:XG ratio of 6:100 (SN.X2) was selected for the preparation of the subsequent DN cryogels, as maintained adequate structural integrity and a suitable porous architecture.

For the DN cryogels, the first XG network was prepared using 6 wt.% XG and an ECH molar ratio of 6:100, followed by the incorporation of 25 wt.% LN as the second network. In contrast to the SN systems, increasing the ECH:OH ratio used for the formation of the second network from 8:100 to 20:100 resulted in a progressive decrease in the amount of LN leached from the DN cryogels. At an ECH ratio of 8:100, DN.XL1 exhibited the highest LN removal, corresponding to 41.24 ± 2.26%. Increasing the ratio to 15:100 reduced the amount of removed LN to 23.12 ± 0.94% for DN.XL2, while a further increase to 20:100 resulted in only 9.10 ± 1.72% LN removal for DN.XL3. Thus, the progressive decrease in LN loss with increasing ECH ratio indicates enhanced stabilization and incorporation of LN within the DN structure. These results suggest that higher cross-linker concentrations favor the formation of a more effectively stabilized second network, thereby limiting the fraction of LN that can be leached during the washing and purification steps.

### 2.2. Characterization of SN and DN Cryogels

#### 2.2.1. FTIR

**Figure 2:**
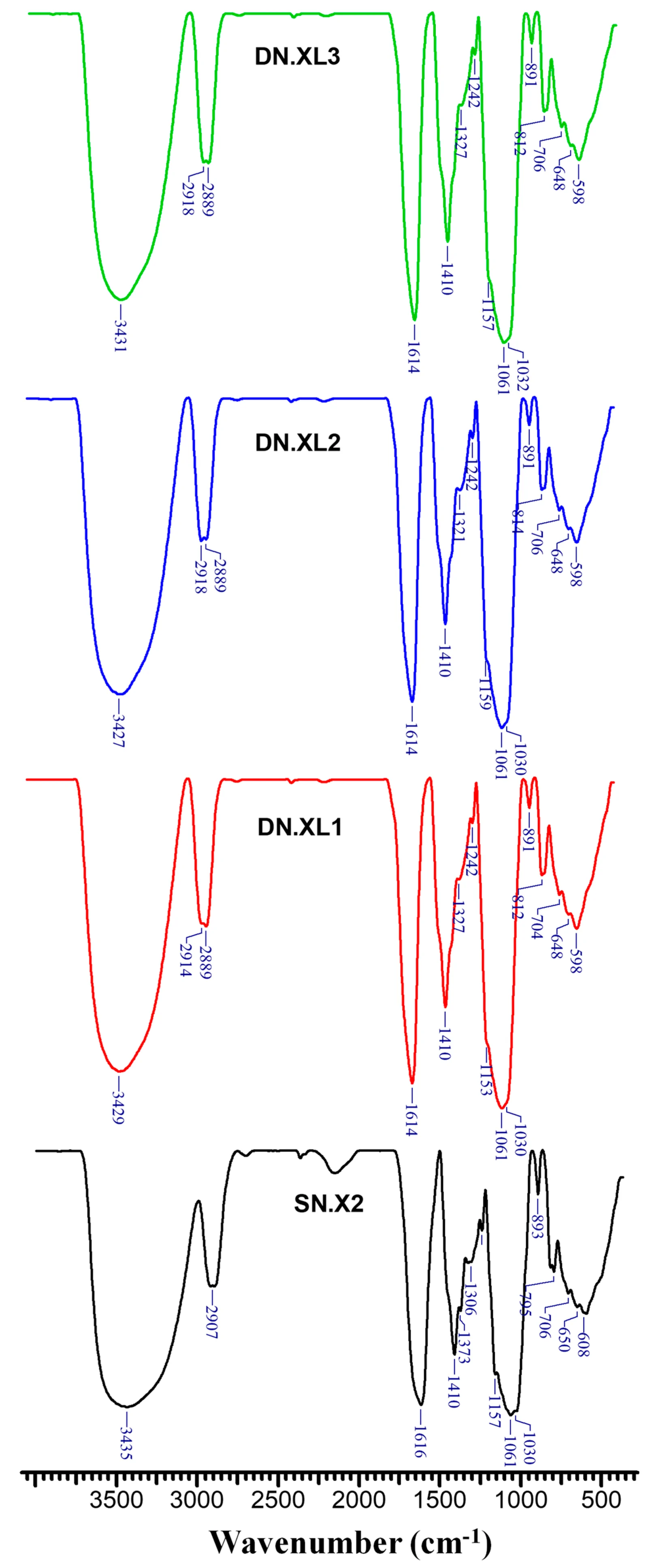
FT-IR spectra of SN and DN cryogels.

FTIR spectroscopy was used to evaluate the chemical structure of the XG-based cryogels and to assess the structural changes associated with ECH-mediated cross-linking and subsequent generation of LN as a second network. The spectra of the SN cryogels (sample SN.X2, Figure 2) and of the DN cryogels (DN.XL1, DN.XL2 and DN.XL3) are presented in Figure 2. The FTIR spectrum of uncross-linked XG is included in Supporting Information as Figure S1.

The FTIR spectra of both SN and DN cryogels show that the characteristic absorption bands of the polysaccharide backbone was preserved, while several changes in band position and intensity, particularly in the C–O/C–O–C region, provide evidence of chemical cross-linking by ECH and network formation.

In the FTIR spectrum of uncross-linked XG (Figure S1, Supporting information) the following absorption bands are present: a broad band at 3560 cm^−1^, which was associated with the stretching vibration of hydroxyl (-OH) groups [29,51,52,53,54,55]. The bands observed at 2920 and 2889 cm^−1^ are attributed to the asymmetric and symmetric stretching vibrations of the -CH_2_ groups, respectively. The absorption bands at 1740 and 1634 cm^−1^ were assigned to the carbonyl (C=O) stretching vibration of acetyl groups and the asymmetric C=O stretching vibration of pyruvate groups, respectively [29,51,52,53,54,55]. The band appearing at 1416 cm^−1^ corresponds to the symmetric stretching vibration of carboxylate (-COO^−^) groups [52,56]. The shoulder around 1161 cm^−1^ and a broad absorption band near 1020 cm^−1^ were attributed to C–O stretching in secondary alcohols and to C–O–C stretching associated with the anhydroglucose (AGU) units, respectively [29,53,54,55].

Following ECH-mediated cross-linking, the main vibrational features of XG were retained in the FTIR spectra of the SN and DN cryogels, indicating preservation of the polysaccharide backbone. Nevertheless, several changes in peak positions and intensities were observed, as follows: the peaks at 3560 cm^−1^ in the FTIR spectrum of uncross-linked XG attributed to the –OH stretching vibration was red-shifted to 3435, 3429, 3427, and respectively 3431 cm^−1^ in the FTIR spectra of SN.X2, DN.XL1, DN.XL2, and DN.XL3 cryogels. The absorption band at 1740 cm^−1^, which is associated with the stretching vibration of the C=O groups in acetyl moieties, exhibited a pronounced reduction in intensity in the cross-linked XG matrices, appearing only as a weak shoulder. This change can be attributed to the hydrolysis of acetyl groups, a reaction that is favored under alkaline conditions [29]. The absorption band at 1634 cm^−1^, attributed to the asymmetric stretching vibration of the C=O groups in pyruvate moieties, red-shifted, appearing at 1616 and 1614 cm^−1^ in the FTIR spectra of the SN and DN cryogels, respectively. Moreover, the characteristic band at 1020 cm^−1^ observed in the spectrum of uncross-linked XG, which is assigned to the stretching vibration of the C–O–C bridge within AGU units, shifted to 1030 cm^−1^ following cross-linking, as observed for both SN and DN cryogels (Figure 2). Additionally, the distinct absorption band at 1061 cm^−1^ in the spectra of both cryogels may indicate the formation or increased contribution of ether (–C–O–C–) and hydroxyl (–OH) groups [29,52,53,54,55,56]. Thus, these spectral modifications provide evidence for the successful occurrence of the ECH-mediated cross-linking reaction. The incorporation of LN as the second network is expected to introduce additional contributions from aromatic and phenolic functionalities. In this regard, a new band at 814 and 812 cm^−1^ was identified in the FTIR spectra of all DN cryogels. This absorption peak can be assigned to the aromatic ring C–H out-of-plane deformation (bending) vibration of substituted benzene rings in LN (specifically related to guaiacyl units or 1,3,4-trisubstituted aromatic rings) [57,58].

The FTIR results are consistent with successful ECH-mediated network formation while indicating that the chemical identity of the XG backbone is largely preserved and that LN remains incorporated within the resulting DN cryogels.

#### 2.2.2. SEM Analysis

**Figure 3:**
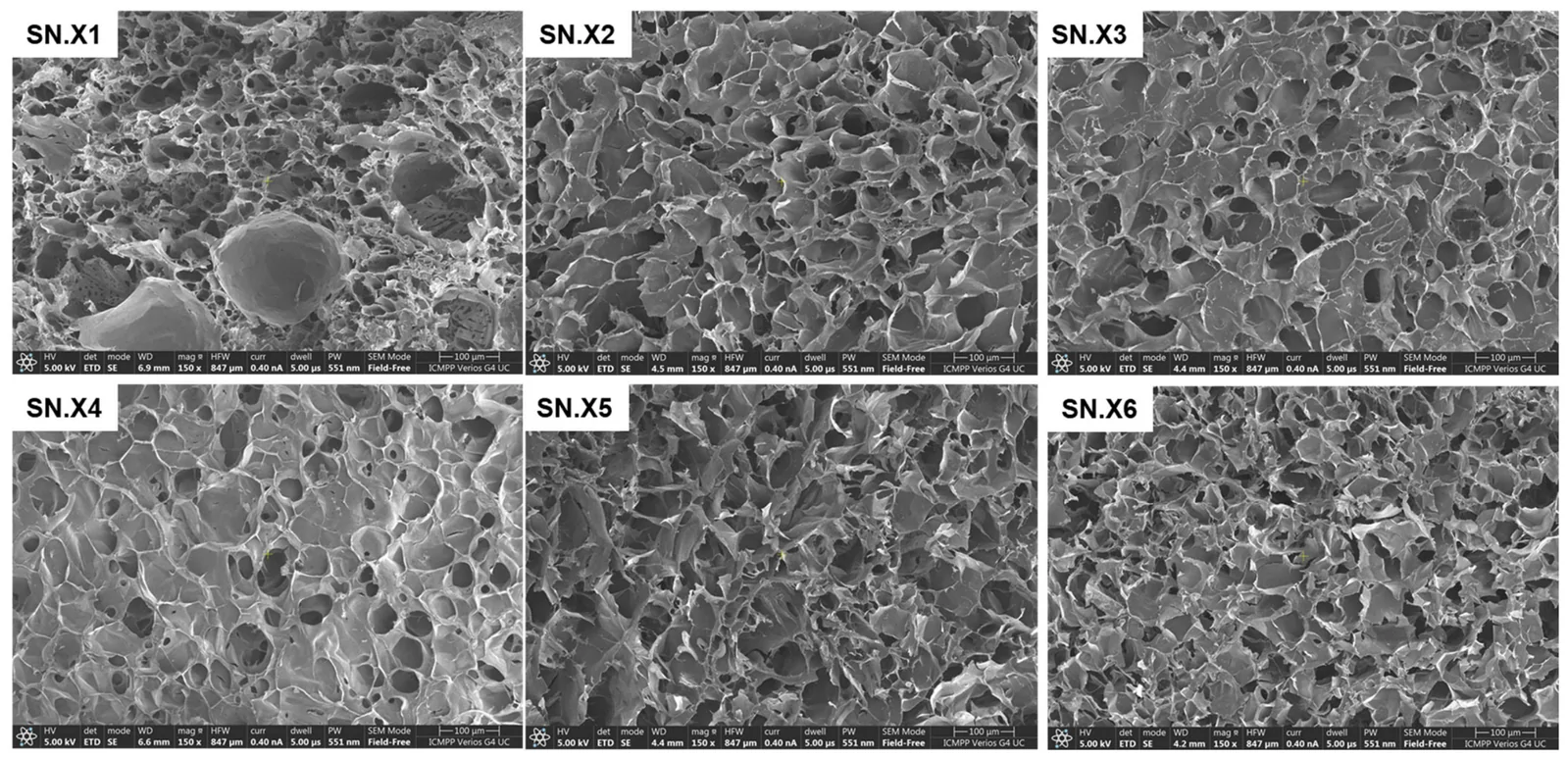
SEM micrographs of the SN cryogels differ by the cross-linker content. Scale bar is 100 μm and mag 150× in all SEM micrographs. The code of samples is as presented in Table 1.

The internal morphology was examined by SEM first for the SN cryogels (Figure 3). All micrographs were obtained at the same magnification (150×) and with a scale bar of 100 µm, allowing for a qualitative comparison of the pore architecture as a function of ECH concentration. As the ECH:XG ratio progressively increased from 3:100 for SN.X1 to 30:100 for SN.X6, changes in morphology in pore geometry, wall morphology, and structural compactness can be observed. The SEM micrographs demonstrate that all samples possess a 3D interconnected porous structure (Figure 3), which confirm the formation of a cryogel network [10,18,22,29,59,60,61].

As Figure 3 shows, at low ECH concentration (sample SN.X1), the network exhibited a highly heterogeneous morphology characterized by large cavities and relatively fragile polymeric walls. Increasing the ECH concentration resulted in the formation of a more interconnected and structurally homogeneous porous network (SN.X2–SN.X4). However, at higher ECH concentrations, a progressive decrease in pore diameter accompanied by an increase in wall thickness became evident for SN.X3 and SN.X4 (Figure 3). Such morphological changes may restrict the penetration and diffusion of the precursors required for formation of the second LN-based network within the preformed XG matrix and may subsequently limit the accessibility to the internal sorption sites. Further increase in ECH concentrations (SN.X5 and SN.X6) generated morphologies more irregular, rough, and locally compact, suggesting excessive cross-linking and further restriction of the pore structure. Considering both the morphological characteristics and the requirements for constructing the subsequent interpenetrating network, SN.X2 was selected as the first network for the subsequent construction of the LN-based second network. SN.X2 provided a relatively homogeneous interconnected porous architecture (Figure 3; Figure S2, Supporting Information), with clearly defined pore walls. This morphology was considered advantageous for facilitating the diffusion and penetration of the LN precursor throughout the XG framework, promoting the formation of a more homogeneous second network. Furthermore, maintaining an open and accessible pore structure was considered important for preserving pathways for subsequent mass transfer and improving the accessibility of active sites during pollutant adsorption.

**Figure 4:**
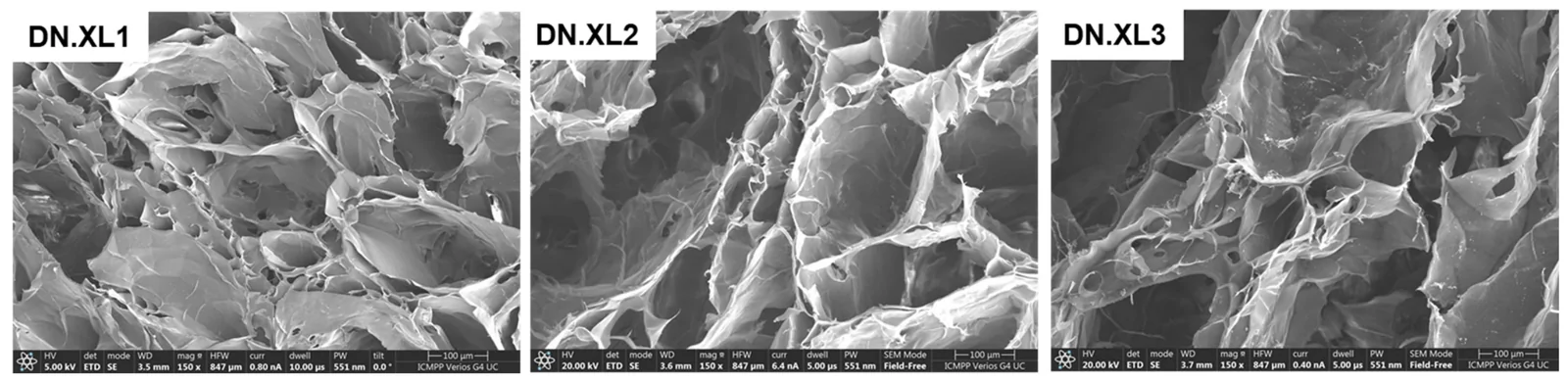
SEM micrographs of the DN cryogels differ by the LN content. Scale bar is 100 μm and mag 150× in all SEM micrographs. The code of samples is as presented in Table 1.

The SEM micrographs for the XG/LN DN cryogels are presented in Figure 4.

As shown in Figure 4, all DN samples retained an interconnected porous architecture, indicating that incorporation of the second LN network did not eliminate the macroporous structure generated during cryogelation. The large, highly interconnected pore structure may facilitate the diffusion of contaminants into the cryogel matrix and may improve their access to the available binding sites.

#### 2.2.3. Swelling Studies

**Figure 5:**
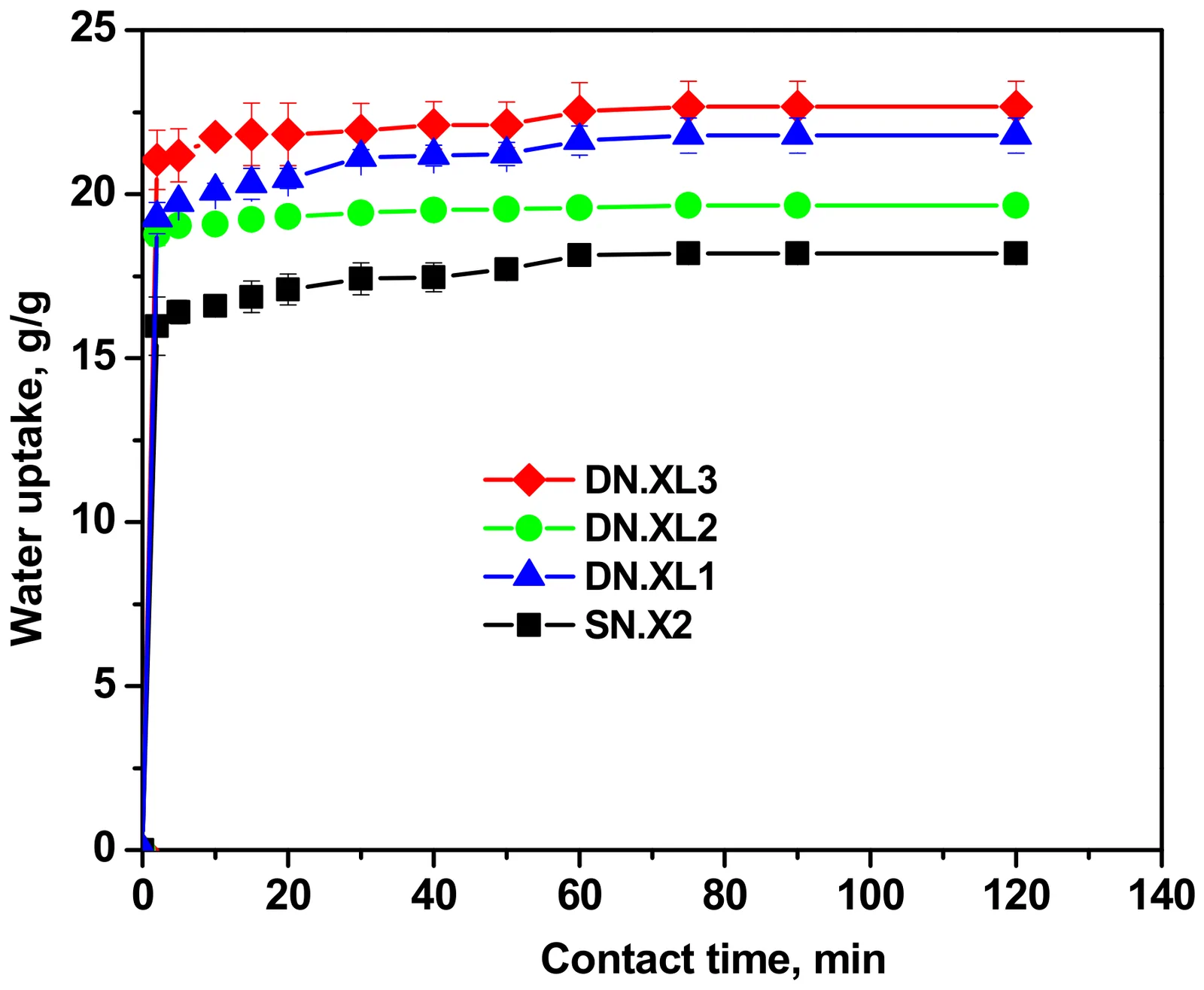
Water uptake (WU) as a function of immersion time for the SN.X2 cryogel and DN cryogels: DN.XL3, DN.XL2, and DN.XL1. WU was determined gravimetrically as described in Section 4.2.7. Data are presented as mean ± standard deviation.

The water uptake (WU, g/g) behavior of the SN and DN cryogels was investigated as a function of immersion time to assess their hydration capacity and WU kinetics. As shown in Figure 5, the values of WU for all cryogels increased in time in two steps until the equilibrium WU was reached. This two-stage behavior is characteristic of highly porous hydrophilic cryogels, in which water can rapidly penetrate the interconnected pore structure during the initial stage, followed by progressive hydration and swelling of the polymeric walls [29,36,62].

The equilibrium WU values varied among the investigated formulations, ranging from 18.19 to 22.66 g/g. The highest water uptake was observed for DN.XL3, which reached an equilibrium value of 22.66 g/g (Figure 5). Compared with SN.X2, used as the reference sample, this value represents an increase of approximately 20%. The enhanced hydration capacity of DN.XL3 suggests that the incorporation of the LN-containing second network did not significantly compromise the water-accessible porosity of the original XG network. Instead, the resulting DN architecture appears to preserve a sufficiently open porous structure, as also evidenced by the SEM micrographs (Figure 4), while providing additional hydrophilic sites and polymer–water interfaces. Similar results were also reported for other SN or DN-based polysaccharide-based sponges [29,36,62].

The differences among the DN formulations further indicate that WU is governed by the combined effects of polymer composition, network organization, and cross-linking conditions. Although DN.XL3 exhibited the highest equilibrium WU, the values did not increase monotonically across DN.XL1–DN.XL3. This behavior suggests that changes in LN incorporation and ECH-mediated network formation may affect the balance between hydrophilic functionality and the accessibility of the porous network. A higher degree of network formation can stabilize the polymer matrix, but excessive restriction of polymer-chain mobility or reduction in accessible free volume could potentially limit swelling [36]. Conversely, incorporation of LN introduced additional water-interacting functionalities without causing blockage of the interconnected pores.

The relatively high hydration capacity of the DN cryogels can also be related to their chemical composition. Both XG and LN contain oxygen-rich functional groups capable of interacting with water through hydrogen bonding. In the case of XG, the abundance of OH groups contributes significantly to its hydrophilic character. Although some OH groups participate in ECH-mediated cross-linking, FTIR analysis indicates that a considerable proportion of the characteristic oxygen-containing functionalities remains after network formation. LN additionally contributes with hydroxyl-containing functionalities, including phenolic groups, which can participate in interactions with water. Thus, the high WU of the DN cryogels can be attributed to the combined contribution of their hydrophilic chemical functionalities and interconnected porous architecture.

DVS measurements further revealed higher water uptake for DN.XL3 than for SN.X2, particularly at higher relative humidity (RH) (Figure S3, Supporting Information). This difference was reflected in the Guggenheim–Andersen–de Boer (GAB) analysis, which yielded monolayer capacities of 0.1216 and 0.0792 g/g and corresponding apparent water-vapor-derived specific surface areas (S_GAB_) of 427.1 and 278.1 m^2^/g for DN.XL3 and SN.X2, respectively. In contrast, the Brunauer–Emmett–Teller (BET) analysis yielded very similar specific surface areas (S_BET_) for both networks, namely 277.6 m^2^/g for DN.XL3 and 283.6 m^2^/g for SN.X2, with corresponding monolayer capacities of 0.0791 and 0.0808 g/g, respectively. Thus, the BET results indicate comparable accessible surface areas for the two networks. The substantially higher GAB-derived area calculated for DN.XL3 should therefore not be interpreted as evidence of a proportionally larger physical surface area, but rather as reflecting its higher water uptake under the higher-relative-humidity conditions used for the GAB analysis. The difference between the GAB- and BET-derived values also reflects the different RH/relative-pressure ranges used for the respective analyses, ~40–81% for GAB analysis and ~5–43% for BET analysis. Overall, these results indicate that water sorption in the XG-based cryogels is governed not only by the accessible surface area but also by the nature and accessibility of sorption sites and by water–polymer interactions.

The morphological observations discussed in Section 2.2.2 provide further support for this interpretation. All DN samples retained a 3D interconnected pore structure despite differences in pore-wall morphology and heterogeneity. Such architecture facilitates water penetration throughout the cryogel structure and provides a large internal surface area accessible to the aqueous phase. The combination of interconnected macropores and hydrophilic polymeric components is therefore favorable for rapid hydration and high equilibrium WU.

The non-monotonic differences among DN.XL1–DN.XL3 demonstrate that hydration is not controlled by LN content or cross-linking degree alone, but rather by the resulting balance between hydrophilic functionality, pore accessibility, and network structure. These characteristics are advantageous for applications requiring rapid interaction with aqueous media and efficient transport of dissolved species through the cryogel matrix.

#### 2.2.4. Mechanical Properties

**Figure 6:**
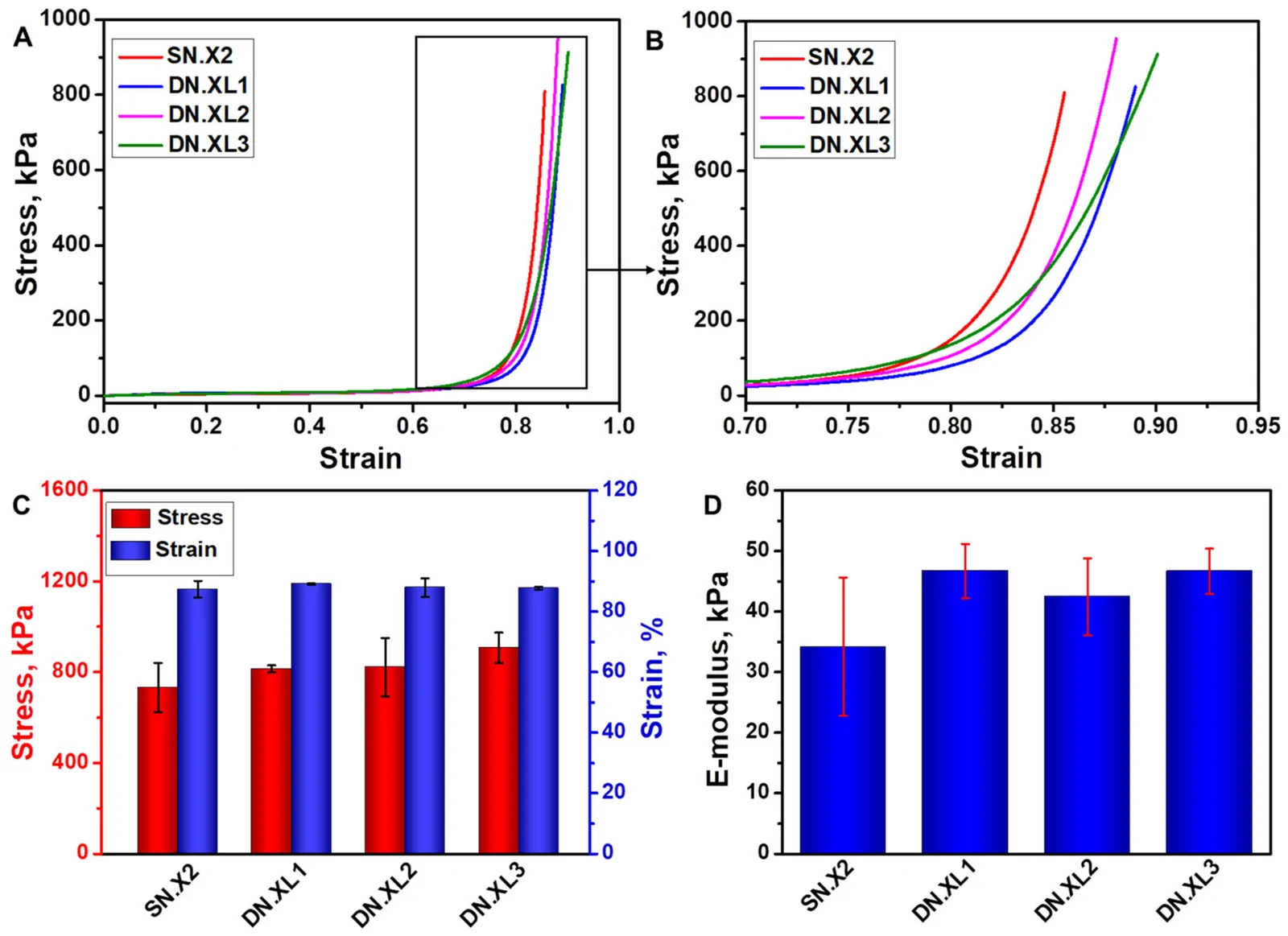
Compressive stress–strain behavior and mechanical parameters of the SN and DN cryogels by applying a force of 50 N: (**A**) representative compressive stress–strain curves of the SN.X2 cryogel and DN.XL1–DN.XL3 cryogels over the complete strain range, with the inset highlighting the high-strain region; (**B**) enlarged view of the nonlinear high-strain region of the stress–strain curves; (**C**) compressive stress and strain corresponds to the maximum applied load; and (**D**) compressive elastic modulus of the investigated cryogels. Values are presented as mean ± standard deviation.

The mechanical behavior of the SN and DN cryogels was evaluated by uniaxial compression testing to determine the effect of incorporating LN as a second network and to assess the influence of the ECH:OH ratio used for formation of the LN-containing network. Typical stress–strain profiles are presented in Figure 6A,B, while the stress, strain, and compressive elastic modulus are displayed in Figure 6C,D.

The stress–strain curves in Figure 6A,B exhibit the characteristic nonlinear response of highly porous cryogels [10,15,62,63,64]. At low strain, all materials show a relatively slight increase in stress, corresponding predominantly to the deformation and progressive collapse of the porous network. As compression proceeds, the stress increases progressively and rises sharply at high strain. This increase is associated with densification of the honeycomb structure, as the pores become progressively collapsed and the solid polymer skeletons come into closer contact. Consequently, the high-strain region provides a particularly sensitive indication of the ability of the network to withstand substantial deformation before mechanical failure or extensive densification. The SN and DN cryogels remained mechanically stable and sustained 87.45, 89.1, 87.9, and respectively 87.75% compression, at a compressive nominal stress of 732.7 kPa, 814.45 kPa, 821.25 kPa, and 907.35 kPa (Figure 6A,C).

A clear improvement in mechanical strength is observed after incorporation of the LN-based second network. This improvement demonstrates that the introduction of LN into the pre-existing XG network contributes to the mechanical reinforcement of the cryogel. It should be noted that during the compression of SN and DN cryogels between the parallel plates of the testing analyzer, the water contained within the cryogel pores was expelled almost completely. This drainage of pore water seems to contribute to the resistance of the cryogels to crack propagation and fracture, particularly at higher strain levels (Figure 6A,B). A similar behavior has been previously reported for chitosan (CS)-based sponges [62], silk fibroin-based cryogels [63] as well as for composite networks based on CS and branched poly(ethyleneimine) [64].

The formation of the DN structure also generates a pronounced increase in the compressive elastic modulus. As shown in Figure 6D, the compressive E-modulus increases from approximately 34 kPa for SN.X2 to 47 kPa for DN.XL1, corresponding to an increase of about 37%. This increase is particularly significant because the elastic modulus describes the resistance of the material to deformation in the initial elastic region. The higher modulus therefore indicates that the LN-containing second network increases the stiffness of the cryogel even before substantial pore collapse occurs. This behavior is consistent with the formation of an additional interconnected network within the XG matrix, which restricts local chain mobility and improves stress transfer throughout the material. Notably, although the compressive strength increased with increasing LN content (Figure 6C), the compressive elastic modulus of the DN formulations remained relatively comparable, with values of 46.69 ± 4.48, 42.44 ± 6.36, and 46.67 ± 3.71 kPa for DN.XL1, DN.XL2, and DN.XL3, respectively (Figure 6D). These results indicate that increasing LN content primarily enhanced the load-bearing capacity/compressive strength of the cryogels rather than modifying their initial elastic stiffness.

The increase in mechanical strength is particularly relevant when considered together with the LN-removal results presented in Table 1. DN.XL1 showed about 41 wt.% LN removal, whereas LN removal decreased to approximately 23 wt.% and 9 wt.% for DN.XL2 and DN.XL3, respectively. Therefore, the samples exhibiting progressively greater LN retention also show an overall tendency toward improved compressive strength. This correlation supports the interpretation that the mechanically active second network is associated with the covalent fixation of LN within the XG matrix. In other words, LN that remains effectively incorporated into the cryogel contributes to the formation of an interconnected structure capable of transmitting compressive loads.

The comparison between SN.X2 and the DN materials therefore demonstrates that the incorporation of LN as a second network provides a significant mechanical reinforcement of the XG cryogel. The increase in compressive strength from approximately 732.7 kPa for SN.X2 to approximately 907.35 kPa for DN.XL3 corresponds to an overall improvement of about 23%, while the modulus increases by about 37% from SN.X2 to the most highly reinforced DN formulations. Importantly, these improvements are achieved while maintaining failure strains close to 90%. The results indicate that the second network increases the resistance of the material to mechanical loading without eliminating its ability to undergo extensive deformation. Thus, the DN.XL3 cryogel showed compressive stress value (at 87.9% strain) of 907.35 kPa, which was greater than 684 kPa (at 76% strain) for CS-based polyelectrolyte complex cryogels [65], 76 kPa (at 70% strain) for macroporous DN cryogels consisting of polyacrylamide and poly(N-isopropylacrylamide) [66], 30.6 kPa (at 26% strain) for cellulose-based superabsorbent hydrogels [67], and 22.76 kPa (at 90% strain) for CS hydrogel scaffolds reinforced with cellulose nanofibers [68].

Moreover, the mechanical behavior can also be correlated with the water uptake results. It is well known that the degree of swelling is an important parameter governing the fracture strain of hydrogels, as the absorbed water can enhance polymer-chain mobility and flexibility within the hydrated network [64,69]. The DN cryogels exhibit higher compressive strength and modulus than the SN cryogels, demonstrating that the second network provides mechanical reinforcement. At the same time, the DN cryogels exhibited high WU values. In the present system, the DN architecture appears to provide reinforcement without eliminating the highly hydrophilic and porous character of the cryogel. The XG/LN networks therefore retain sufficient free volume and accessible hydrophilic groups to accommodate substantial amounts of water while providing improved mechanical integrity.

From a structural perspective, the observed behavior can be explained by the formation of a hierarchical load-bearing architecture. The first XG network provides the primary porous scaffold, while the LN-based second network occupies and reinforces the existing network structure. ECH-mediated cross-linking of the LN phase increases its stability within the XG matrix, allowing stress to be distributed through both networks rather than being carried exclusively by the XG framework. At higher ECH ratios, the increased retention and stabilization of LN further improve the integrity of the composite network, which is reflected most clearly in the increased maximum compressive stress of DN.XL3. Thus, the mechanical results demonstrate that DN formation is an effective strategy for simultaneously increasing the stiffness and compressive strength of XG cryogels while retaining high deformability. The results also show that the mechanical response cannot be attributed solely to the amount of cross-linker. Instead, it is governed by the combined effects of second-network formation, LN retention, covalent network connectivity and the porous cryogel morphology. Among the investigated formulations, DN.XL3, prepared using the highest ECH:OH ratio, exhibited the highest compressive strength and a high elastic modulus while retaining a strain comparable to the other DN formulations. These characteristics identify the highly cross-linked XG/LN DN architecture as the most mechanically reinforced formulation among the samples investigated.

### 2.3. Potential Application of XG/LN-Based Networks

#### 2.3.1. Antimicrobial Cryogel-Based Materials

**Figure 7:**
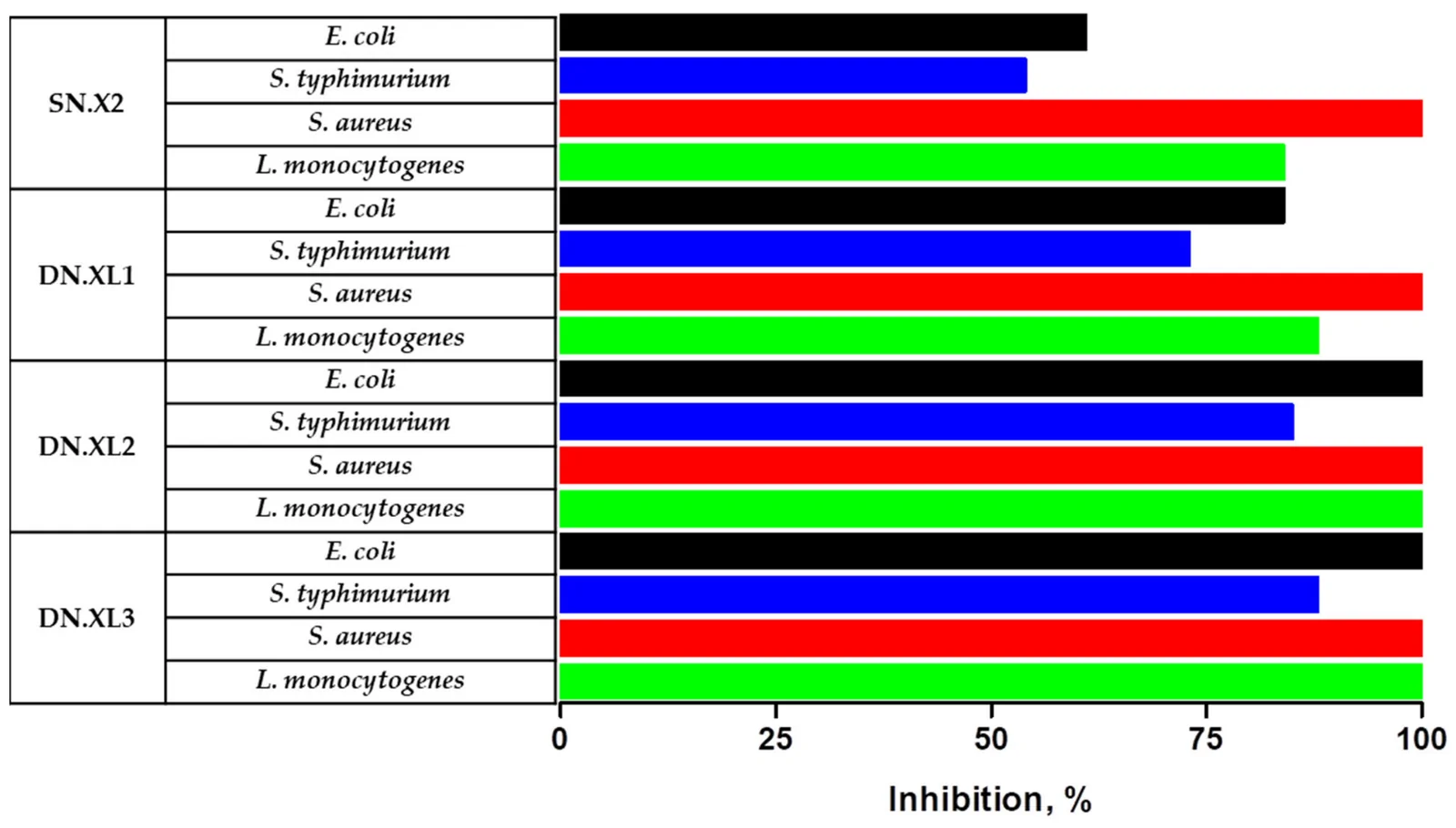
Antimicrobial activity of SN.X2, DN.XL1, DN.XL2, and DN.XL3 against four bacterial strains, expressed as percentage inhibition. Black bars represent *E. coli*, blue bars represent *S. Typhimurium*, red bars represent *S. aureus*, and green bars represent *L. monocytogenes* bacterial species.

XG, LN, and materials based on them have long been under investigation for their excellent antibacterial properties [28,62,70,71,72,73,74,75,76,77,78]. Therefore, the antimicrobial activity of SN and DN cryogels was evaluated towards two Gram-negative (*E. coli*, *S. typhimurium*) and two Gram-positive (*L. monocytogenes*, *S. aureus*) bacterial strains (Figure 7 and Figure S4, Supporting Information).

As shown in Figure 7 and Figure S4, all XG-based cryogels exhibited considerable antimicrobial activity against the four tested bacterial strains, although the extent of inhibition depended on both the bacterial species and the cryogel composition. Among the investigated samples, SN.X2 exhibited the lowest overall antimicrobial activity, whereas the DN cryogels, particularly DN.XL2 and DN.XL3, showed the most pronounced and broad-spectrum inhibitory effects. This trend indicates that the incorporation of the LN as the second network significantly enhanced the antimicrobial performance of the XG-based cryogels. The antimicrobial properties of LN are associated with its inherent role in plant defense against pathogenic microorganisms [70,71]. Within plant tissues, LN contributes to the protection of structural carbohydrates by limiting their degradation and reducing microbial invasion by bacteria and fungi [70]. Owing to these natural protective properties, technical lignins obtained from lignocellulosic biomass have attracted considerable interest in biological and biomedical applications, where they have been investigated as potential antibacterial, antifungal, and antiviral agents [70,71,72,73,74,75,76,77,78].

Among the tested microorganisms, *S. aureus* was the most susceptible strain (Figure 7), 100% inhibition being observed for all four samples. This result indicates that no viable colonies were detected under the applied experimental conditions in the corresponding recovered samples. *L. monocytogenes* also exhibited a high susceptibility, with inhibition values of approximately 84% for SN.X2 and 88% for DN.XL1, increasing to complete inhibition for DN.XL2 and DN.XL3. The progressive increase in inhibition observed for this strain suggests that the structural and compositional modifications introduced in the DN.XL2 and DN.XL3 systems resulted in a more effective antimicrobial response.

A similar trend was observed for the Gram-negative bacterium *E. coli*, although its response was more dependent on the cryogel composition. The calculated inhibition increased from approximately 61% for SN.X2 to 84% for DN.XL1 and reached nearly 100% for both DN.XL2 and DN.XL3. The substantially higher inhibition values obtained for the DN cryogels suggests that the incorporation of LN into the XG network contributes to the observed antibacterial response.

The higher inhibition observed for the LN-containing DN cryogels, may be related to the chemical functionality of LN. LN contains phenolic and other oxygen-containing functional groups that have been associated with interactions with bacterial cell envelopes and with antimicrobial effects reported for different LN preparations [71,72]. However, the antimicrobial behavior of LN is strongly dependent on its botanical origin, extraction/isolation procedure, molecular structure, functional-group composition, and concentration, as well as on the characteristics of the target microorganism. For example, Dong et al. reported that kraft LN obtained from corn exhibited pronounced antibacterial activity against the Gram-positive strains *Listeria monocytogenes* and *Staphylococcus aureus*, while showing limited activity toward Gram-negative bacteria and bacteriophages [74]. In contrast, Lourençon et al. demonstrated that kraft LN derived from eucalyptus was effective against a broader range of microorganisms, including the Gram-positive species *Bacillus cereus* and *Staphylococcus aureus*, as well as the Gram-negative strains *Pseudomonas aeruginosa*, *Escherichia coli*, and *Salmonella enteritidis* [73].

Beyond its direct application as an antimicrobial material, LN has also been incorporated into multifunctional systems to impart antibacterial properties. For instance, Ritcher et al. developed a system in which LN was deposited around silver nanoparticles, providing effective inhibition of *Staphylococcus aureus* and *Escherichia coli* while limiting the release of environmentally undesirable silver ions [75]. Similarly, several studies have investigated LN-containing polymer films in which LN acts as functional filler. LN–polymer composites have demonstrated antibacterial activity against a variety of bacterial species, highlighting the potential of lignin as an antimicrobial component in polymeric materials [76,77,78].

In contrast, *S. Typhimurium* exhibited the lowest calculated inhibition values, increasing from approximately 54% for SN.X2 to 73% for DN.XL1, 85% for DN.XL2, and 88% for DN.XL3. Thus, although *S. Typhimurium* displayed a greater resistance to the cryogels than the other tested strains, its inhibition progressively increased along the SN.X1–DN.XL3 series. The lower susceptibility of this strain may be related to differences in cell-envelope characteristics and other physiological mechanisms that can affect the interaction of antimicrobial compounds with bacterial cells.

In conclusion, the results presented in Figure 7 and Figure S4 demonstrate that the tested XG-based cryogels were capable of reducing the number of recoverable viable bacterial cells under the experimental conditions employed, with the magnitude of inhibition depending on both bacterial strain and cryogel composition. The DN formulations generally exhibited higher inhibition values than SN.X2, particularly for *L. monocytogenes*, *E. coli*, and *S. Typhimurium*. *S. aureus* showed no detectable recoverable colonies for all investigated formulations, whereas *S. Typhimurium* exhibited the lowest calculated inhibition. These findings suggest that incorporation of the LN-based second network may enhance the bacterial inhibition observed for the XG cryogels.

#### 2.3.2. Adsorbent Materials

##### SNs as Sorbents for Cu^2+^ Ions

As a first step, the influence of the sorbent dose on the sorption capacity (q_e_, mg/g) and removal efficiency (RE, %) of Cu^2+^ ions by the SNs was investigated by varying the sorbent mass from 0.01 to 0.035 g. As shown in Figure S5A (Supporting Information), the RE increased to approximately 83% as the sorbent dose increased from 0.01 to 0.02 g and remained nearly constant upon further increasing the sorbent dose to 0.035 g for the SN.X2 cryogels. In contrast, q_e_ decreased monotonically with increasing sorbent dose. This behavior can be attributed to the increase in the number of available active sites with increasing sorbent dose, resulting in the same amount of Cu^2+^ ions being distributed over a larger number of active sites.

Based on the sorbent dose experiments, a sorbent mass of 0.02 g was selected for the kinetic study. As Figure S5B (Supporting Information) shows, Cu^2+^ sorption was very rapid during the initial stage, followed by a gradual decrease in the sorption rate until equilibrium was reached. For the SN.X2 cryogels, the rapid initial uptake can be attributed to the high availability of accessible active sites and the large concentration gradient between the solution and the sorbent. As these sites became progressively occupied, the sorption rate decreased, and reached equilibrium in about 250 min.

The sorption kinetics of SN.X2 sorbents were evaluated using the pseudo-first-order (PFO) and pseudo-second-order (PSO) models, and the corresponding calculated parameters are presented in Table S1 (Supporting Information). For SN.X2 cryogels, the PFO model provided a better description of the experimental kinetic data than the PSO model. The PFO model gave a calculated equilibrium capacity of 85.71 mg/g, which is relatively close to the experimental value, with an R^2^ of 0.979 and a χ^2^ value of 22.49. In comparison, the PSO model yielded a higher calculated capacity of 99.81 mg/g, together with a lower R^2^ (0.942) and a higher χ^2^ value (61.66). The good fit of the PFO model suggests that the rate of adsorption of SN.X2 may be predominantly associated with the availability of adsorption sites and the rapid uptake of the adsorbate during the early stages of the process.

**Figure 8:**
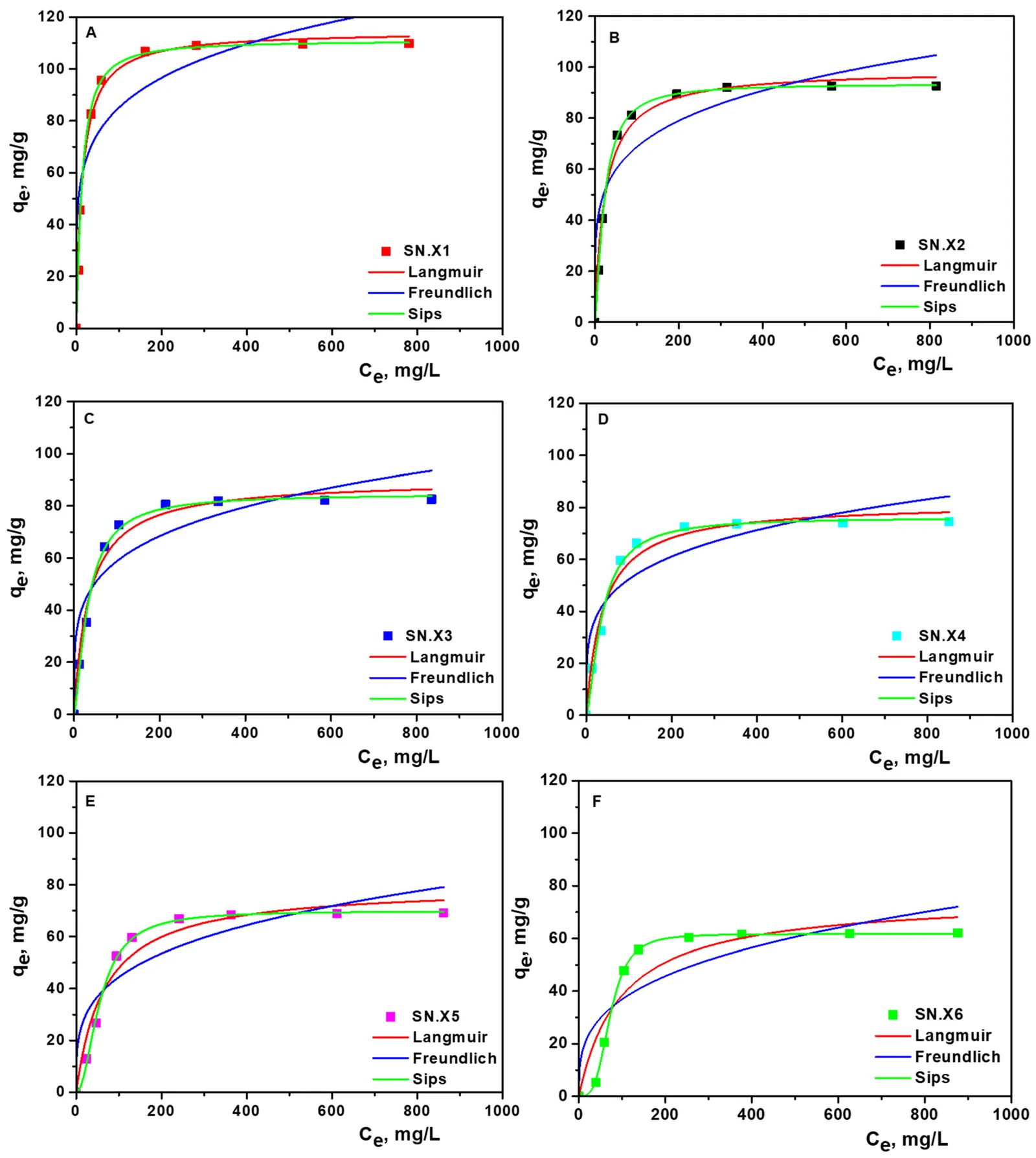
Equilibrium sorption isotherms of Cu^2+^ ions onto single-network XG-based (SN.X) gels. Equilibrium sorption capacities (q_e_) as a function of equilibrium Cu^2+^ concentration (C_e_) are shown for (**A**) SN.X1, (**B**) SN.X2, (**C**) SN.X3, (**D**) SN.X4, (**E**) SN.X5, and (**F**) SN.X6. The experimental data are represented by colored symbols, while the solid lines correspond to the Langmuir (red), Freundlich (blue), and Sips (green) isotherm models. The (q_e_) and (C_e_) values are expressed in mg/g and mg/L, respectively.

The equilibrium sorption isotherms shown in Figure 8 for SN-based sorbents indicated that the sorption capacity (q_e_) increased rapidly with increasing equilibrium Cu^2+^ concentration (C_e_) at the initial stage and subsequently approached a plateau. This characteristic profile indicates that the gels possess a finite number of Cu^2+^-binding sites that are progressively occupied as the metal-ion concentration increases. The initial portion of the isotherms indicates a high availability and affinity of the accessible sorption sites at relatively low Cu^2+^ concentrations, whereas the progressive decrease in slope at higher concentrations is associated with increasing occupation of these sites and eventual saturation. The experimental maximum sorption capacities (q_m_,_exp_) varied among the investigated cryogels, decreasing in the order SN.X1 (109.81 mg/g) > SN.X2 (92.74 mg/g) > SN.X3 (82.54 mg/g) > SN.X4 (74.53 mg/g) > SN.X5 (69.18 mg/g) > SN.X6 (62.13 mg/g). Thus, SN.X1 exhibited the highest Cu^2+^ uptake, whereas SN.X6 showed the lowest sorption capacity, which can be attributed to its higher degree of cross-linking. The approximately 77% higher sorption capacity of SN.X1 compared with SN.X6 indicates that a less cross-linked network possesses a higher content of available functional groups for binding Cu^2+^ ions.

Among the numerous equations proposed to describe equilibrium data, the Langmuir Equation (1), Freundlich Equation (2), and Sips Equation (3) isotherms are the most frequently employed [79,80,81,82]. Therefore, the experimental results obtained in this study were evaluated using these three approaches and the values of the equilibrium isotherm parameters for Cu^2+^ sorption onto SNs are presented in Table 2.

(1) $$q_{e}=\frac{q_{m}K_{L}C_{e}}{1+K_{L}C_{e}}$$

(2) $$q_{e}=K_{F}C_{e}^{1/n}$$

(3) $$q_{e}=\frac{a_{S}C_{e}^{1/n}}{1+a_{S}C_{e}^{1/n}}$$

where q_e_ (mg/g) is the sorbed amount at equilibrium; C_e_ (mg/L) is the equilibrium concentration; q_m_ (mg/g) is the maximum sorbed amount; *K_L_* (L/mg) is the Langmuir adsorption constant; K_F_ is the Freundlich constant; *a_S_* (L/mg) is the Sips adsorption constant; and 1/n is the parameter related to surface heterogeneity. Values between 0 and 1 for 1/n parameter are associated with favorable uptake, 1/n = 1 indicates a reversible process, while values above 1 suggest an unfavorable interaction.

The calculated isotherm parameters provide strong evidence that the Sips model provides the most appropriate description of Cu^2+^ sorption by the investigated gels. The Sips model yielded high coefficients of determination, with (R^2^) values between 0.992 and 1.000. These values are consistently higher than those obtained from the Langmuir model (0.992–0.866) and the Freundlich model (0.858–0.737). Moreover, the corresponding (*χ*^2^) values for the Sips model were very low, ranging from 0.276 to 7.721, compared with 14.708–89.230 for the Langmuir model and 112.539–253.833 for the Freundlich model. The superiority of the Sips model is also demonstrated by the close agreement between its calculated maximum sorption capacities and the experimental values (Table 2). The excellent performance of the Sips model indicates that Cu^2+^ sorption by these XG-based cryogels is best described as occurring on a heterogeneous sorption surface with a finite saturation capacity. From an application perspective, the relatively high Cu^2+^ uptake capacities observed for the materials, particularly SN.X1 and SN.X2, indicate their potential for removing Cu^2+^ from aqueous systems.

##### DNs as Sorbents for Cu^2+^ Ions

The effect of sorbent dose on the q_e_ (mg/g) and RE (%) was also investigated for DNs. A similar behavior was observed for DN cryogels (Figure S6A, Supporting Information). However, the RE values increased substantially with increasing sorbent dose, from 73.05% at 0.01 g to 93.92% at 0.02 g, after which only minor changes were observed. The kinetic behavior of DN.XL3 also exhibited a rapid initial adsorption stage followed by a slower approach to equilibrium (Figure S6B, Supporting Information). The experimental equilibrium adsorption capacity was 91.92 mg/g, which was higher than that obtained for SN.X2, indicating a greater adsorption capacity of the DN.XL3 sorbent toward Cu^2+^ ions under the investigated conditions. Similarly to SN.X2, the PFO model provided the better fit to the DN.XL3 kinetic data (Table S1, Supporting Information). The PFO model resulted in a calculated equilibrium capacity of 93.13 mg/g, which is very close to the experimental value of 91.92 mg/g. Moreover, the PFO model exhibited a high R^2^ value of 0.993 and a low χ^2^ value of 7.54. In contrast, the PSO model gave a higher calculated capacity of 102.61 mg/g, with a lower R^2^ of 0.970 and a higher χ^2^ value of 9.95. Thus, based on the combined criteria of R^2^, χ^2^, and the agreement between calculated and experimental capacities, the PFO model more accurately describes the kinetic behavior of DN.XL3.

**Figure 9:**
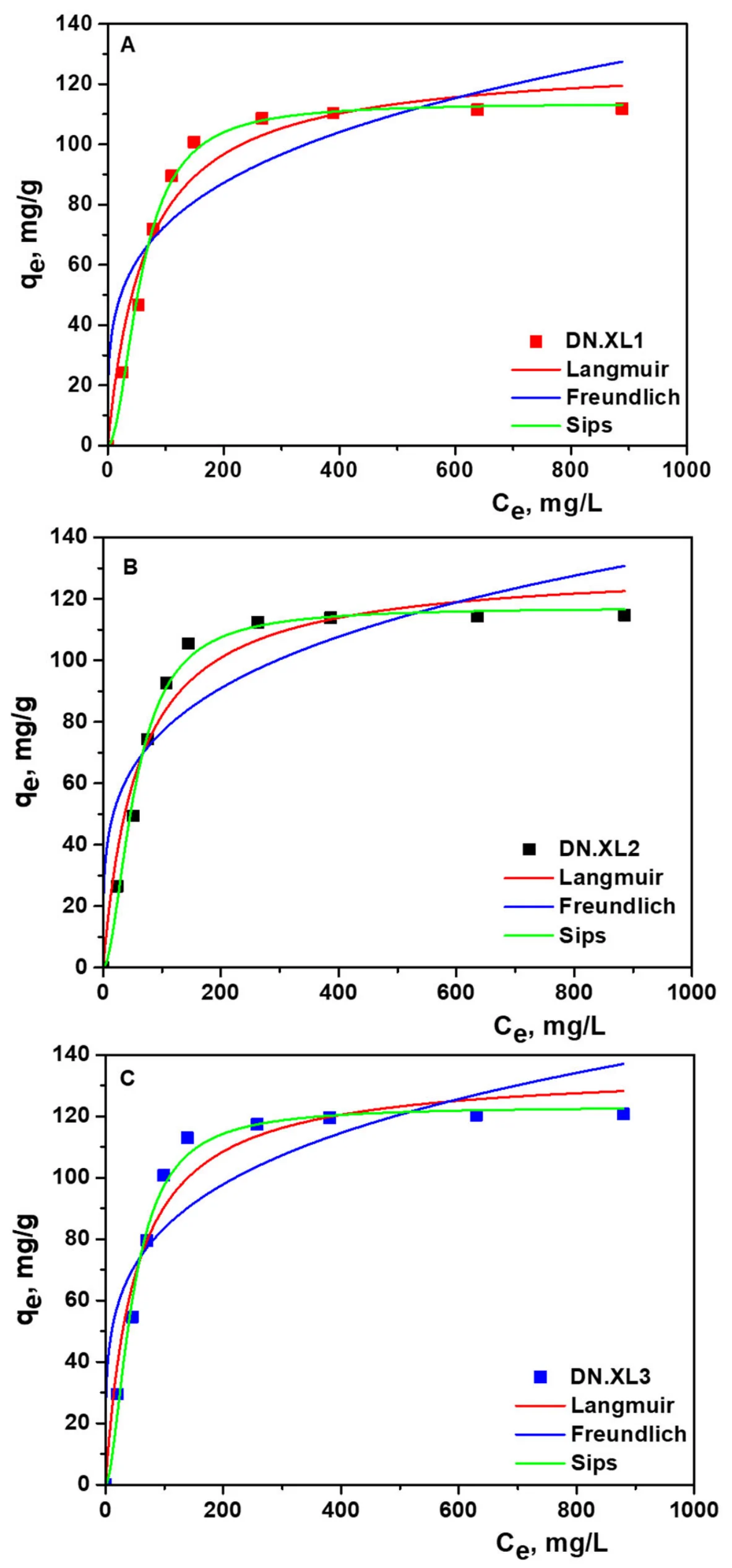
Equilibrium sorption isotherms of Cu^2+^ ions onto DN XG-based cryogels. The equilibrium sorption capacity (q_e_) as a function of equilibrium Cu^2+^ concentration (C_e_) is presented for (**A**) DN.XL1, (**B**) DN.XL2, and (**C**) DN.XL3. Experimental equilibrium data are represented by colored square symbols, whereas the solid curves represent the fitted Langmuir (red), Freundlich (blue), and Sips (green) isotherm models. The (C_e_) and (q_e_) values are expressed in mg/L and mg/g, respectively.

The equilibrium sorption behavior of Cu^2+^ ions onto the DN XG-based gels DN.XL1, DN.XL2, and DN.XL3 is presented in Figure 9. All three gels exhibited a typical nonlinear sorption profile, characterized by a pronounced increase in (q_e_) at low equilibrium Cu^2+^ concentrations (C_e_), and followed by a gradual reduction in the slope and the development of a quasi-plateau at higher concentrations. This behavior indicates a high availability of Cu^2+^-binding sites at low concentrations and their progressive occupation as the metal ion concentration increases.

The experimentally determined maximum sorption capacities (q_m,exp_) were 111.87, 114.73, and 120.84 mg/g for DN.XL1, DN.XL2, and DN.XL3, respectively. Thus, the equilibrium Cu^2+^ uptake increased in the order DN.XL1 < DN.XL2 < DN.XL3, with DN.XL3 exhibiting the highest capacity.

The equilibrium data were fitted using the Langmuir, Freundlich, and Sips isotherm models to clarify the nature of the sorption process. The fitting parameters summarized in Table 3 demonstrate that the Sips model provides the best overall description of Cu^2+^ sorption for all DN cryogels. The Sips model yielded coefficients of determination (R^2^) of 0.994, 0.992, and 0.991 for DN.XL1, DN.XL2, and DN.XL3, respectively, together with lower (*χ*^2^) values of 9.715, 14.358, and 17.695, respectively. In comparison, the Langmuir model produced (R^2^) values of 0.956–0.962 and (*χ*^2^) values of 72.875–73.545, whereas the Freundlich model gave lower (R^2^) values of 0.838–0.840 and much higher (*χ*^2^) values of 268.622–303.459.

The calculated maximum sorption capacities obtained from the Sips model were in close agreement with the corresponding experimental capacities. Moreover, the Sips model reproduced the same capacity sequence as the experimental data, namely DN.XL3 > DN.XL2 > DN.XL1. This close correspondence further supports the suitability of the Sips equation for describing the equilibrium behavior of the DN cryogels.

The fitting of the Sips model is consistent with the physical characteristics of the DN cryogels. The enhanced performance of the DN cryogels may arise from improved structural organization and accessibility of the sorption domains rather than simply from an increase in the total number of chemical binding groups. In hydrogel sorbents, swelling and network architecture strongly influence the diffusion of metal ions and the accessibility of internal functional groups [10,14,15].

The equilibrium results demonstrate that the DN cryogels possess high and concentration-dependent Cu^2+^ sorption capacities, with DN.XL3 showing the highest experimental uptake (120.84 mg/g). Statistical evaluation of the three isotherm models clearly identifies the Sips model as the most appropriate model. The results indicate that Cu^2+^ sorption is governed by a heterogeneous distribution of binding sites together with finite saturation capacity. Compared with the SN cryogels, the higher capacities of the DN cryogels further demonstrate that the DN strategy is an effective approach for improving the equilibrium Cu^2+^ sorption performance of XG-based hydrogel systems.

The influence of potentially competing divalent cations on Cu^2+^ sorption was also evaluated using Mg^2+^ and Ca^2+^ as representative competing ions. The DN.XL3 sorbent exhibited a Cu^2+^ sorption capacity of 120.84 mg/g in deionized water. In the presence of Mg^2+^ and Ca^2+^, the Cu^2+^ sorption capacity decreased only slightly to 117.82 and 118.79 mg/g, respectively. These values correspond to a decrease of approximately 2.5% for Mg^2+^ and 1.7% for Ca^2+^ compared with the sorption in deionized water. The relatively small decrease in Cu^2+^ uptake indicates that the presence of these competing divalent ions had only a limited effect on the Cu^2+^ sorption performance of DN.XL3. This behavior suggests that the sorbent exhibits a certain degree of selectivity toward Cu^2+^ under the investigated conditions, despite the presence of other divalent cations at comparable concentrations.

##### Desorption and Reusability Studies

**Figure 10:**
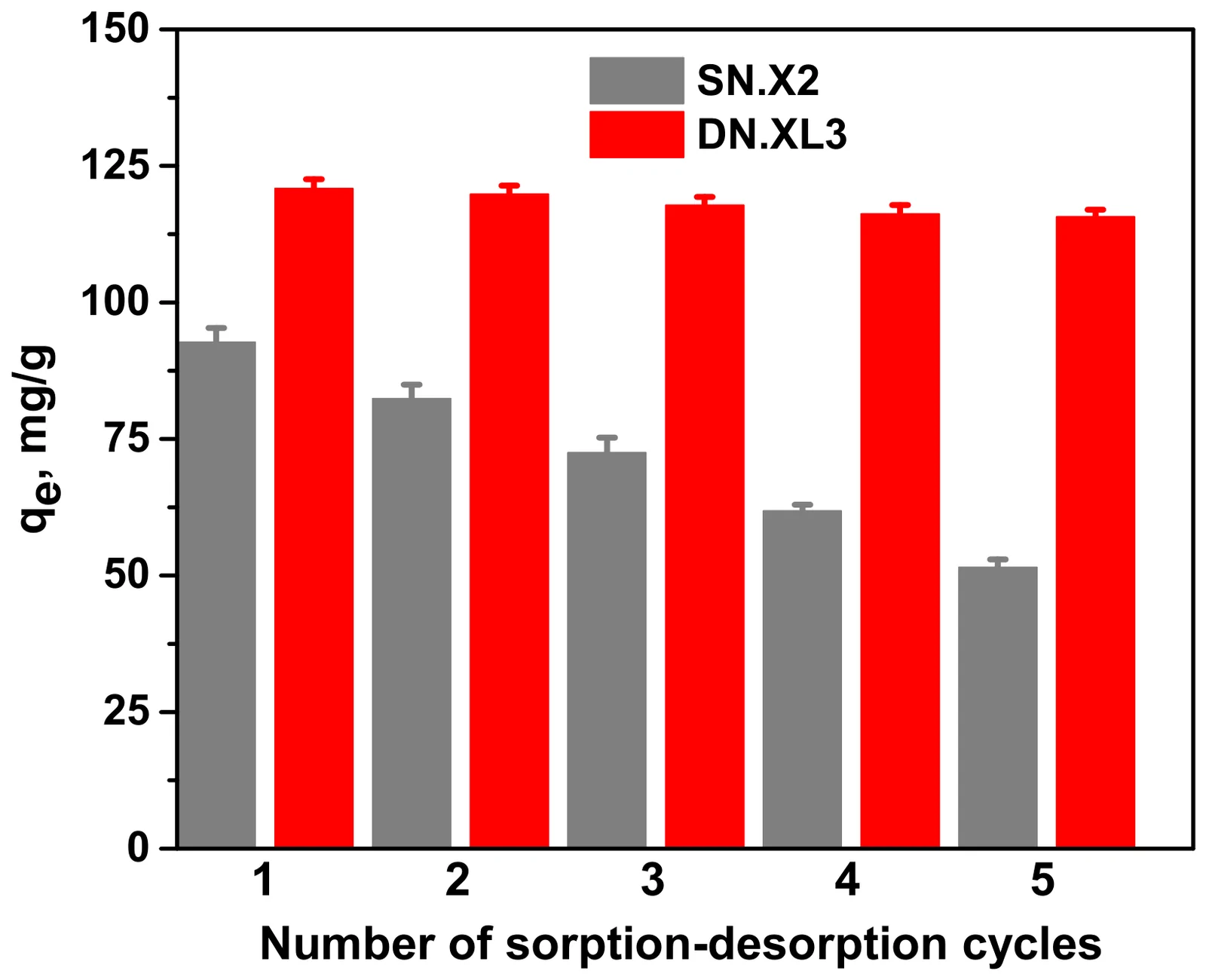
Sorption capacity of Cu^2+^ ions as a function of the number of sorption-desorption cycles. Sorption conditions: sorbent dose = 20 mg; pH = 5.5; V = 0.01 L.

The reusability of the SN.X2 and DN.XL3 sorbents was evaluated over five consecutive sorption–desorption cycles (Figure 10).

As Figure 10 shows, the sorption performance of SN.X2 towards Cu^2+^ ions progressively decreased with increasing the number of sorption-desorption cycles, indicating a gradual loss of accessible sorption sites and/or partial alteration of the SN.X2 structure during repeated sorption–desorption processes. In contrast, the DN.XL3 sorbent exhibited relatively constant sorption capacity over the five cycles. The absence of an abrupt decrease in either sorption or desorption performance suggests that the interpenetrating DN architecture provides enhanced structural integrity during repeated sorption-desorption cycles. This enhanced recyclability is particularly relevant for practical pollutant removal applications, as it can reduce the amount of adsorbent required and minimize the generation of spent sorbent waste.

#### 2.3.3. Cu^2+^-Loaded SN or DN Cryogels as Sorbents of Sulfate Ions

An important aspect of the present study is the evaluation of the sorption properties of the XG-based networks after their loading with Cu^2+^ ions. Rather than considering the Cu^2+^-loaded materials as exhausted sorbents, their ability to subsequently interact with sulfate ions (SO_4_^2−^) was investigated (Table 4).

The results presented in Table 4 demonstrates that the Cu^2+^-loaded networks retain considerable sorption activity toward SO_4_^2−^, revealing a potential sequential or dual-function application in which the same polymeric material can first capture Cu^2+^ and subsequently participate in SO_4_^2−^ removal.

SO_4_^2−^ sorption was evaluated using initial SO_4_^2−^ concentrations (C_i_) of 400 and 1000 mg/L after the networks had been previously loaded with Cu^2+^ at initial concentrations of 500 and 1000 mg/L.

For the SN Cu^2+^-loaded cryogel composites (SN/Cu^2+^), the SO_4_^2−^ sorption capacities ranged from 172.43 to 694.53 mg/g, depending on the initial Cu^2+^ and SO_4_^2−^ concentrations. At an initial Cu^2+^ concentration of 500 mg/L, increasing the SO_4_^2−^ concentration from 400 to 1000 mg/L increased (q_e_) from 172.43 to 508.96 mg/g, while the SO_4_^2−^ removal efficiency increased from 43.1% to 50.9%. When the previous Cu^2+^ loading was increased to 1000 mg/L, the corresponding sulfate capacities were 201.84 and 694.53 mg/g at initial SO_4_^2−^ concentrations of 400 and 1000 mg/L, respectively, corresponding to SO_4_^2−^ removal efficiencies of 50.5% and 69.5%.

The DN composite cryogels (DN/Cu^2+^) exhibited an even more pronounced SO_4_^2−^ sorption performance. For DN.XL1/Cu^2+^, the SO_4_^2−^ sorption capacity increased from 216.92 to 594.36 mg/g when the initial SO_4_^2-^ concentration increased from 400 to 1000 mg/L at a Cu^2+^ loading concentration of 500 mg/L. The corresponding SO_4_^2−^ removal efficiencies were 54.2% and 59.4%, respectively. When the previous Cu^2+^ loading concentration was increased to 1000 mg/L, the sulfate capacities increased to 247.83 and 751.83 mg/g, corresponding to removal efficiencies of 62.0% and 75.2%.

An even higher SO_4_^2−^ uptake was observed for DN.XL2/Cu^2+^. At a previous Cu^2+^ loading concentration of 500 mg/L, SO_4_^2−^ capacities of 243.84 and 617.48 mg/g were obtained at initial SO_4_^2−^ concentrations of 400 and 1000 mg/L, respectively. The corresponding SO_4_^2−^ removal efficiencies were 61.0% and 61.7%. Increasing the previous Cu^2+^ loading concentration to 1000 mg/L further increased the SO_4_^2−^ capacities to 285.76 and 796.54 mg/g, corresponding to removal efficiencies of 71.4% and 79.7%, respectively.

DN.XL3/Cu^2+^ exhibited the highest overall SO_4_^2−^ sorption performance among the investigated cryogels. At a previous Cu^2+^ loading concentration of 500 mg/L, the SO_4_^2−^ sorption capacities were 258.43 and 635.29 mg/g at initial SO_4_^2−^ concentrations of 400 and 1000 mg/L, respectively, corresponding to removal efficiencies of 64.6% and 63.5%. At the higher previous Cu^2+^ loading of 1000 mg/L, the capacities increased further to 319.82 and 812.53 mg/g, with SO_4_^2−^ removal efficiencies of 80.0% and 81.3%, respectively. Thus, DN.XL3/Cu^2+^ composites exhibited the highest SO_4_^2−^ capacity and the highest removal efficiency among all materials and experimental conditions investigated.

Therefore, both the sorption capacity and removal efficiency generally followed the sequence: SN.X2/Cu^2+^ < DN.XL1/Cu^2+^ < DN.XL2/Cu^2+^ < DN.XL3/Cu^2+^. This systematic trend is consistent with the superior structural and sorption characteristics of the DN cryogels observed during the initial Cu^2+^ sorption experiments. In particular, DN.XL3 previously exhibited the highest Cu^2+^ sorption capacity among the DN cryogels, and it also displayed the highest subsequent SO_4_^2−^ uptake. This correspondence suggests that the network architecture plays a central role in controlling not only the initial metal ion sorption step but also the subsequent interaction of the Cu^2+^-loaded network with SO_4_^2−^.

The effect of the previous Cu^2+^ loading is also noteworthy. For most conditions, increasing the initial Cu^2+^ concentration from 400 to 1000 mg/L resulted in a clear increase in SO_4_^2−^ sorption capacity. This effect was particularly pronounced at an initial SO_4_^2−^ concentration of 1000 mg/L. For example, SO_4_^2−^ uptake by DN.XL3 increased from 635.29 to 812.53 mg/g when the previous Cu^2+^ loading concentration increased from 400 to 1000 mg/L. Similarly, DN.XL2 increased from 617.48 to 796.54 mg/g, while DN.XL1 increased from 594.36 to 751.83 mg/g. The single-network material showed the same general tendency, increasing from 508.96 to 694.53 mg/g.

The selectivity of the DN.XL3/Cu^2+^ sorbent toward SO_4_^2−^ was further investigated in the presence of several competing anions, namely NO_3_^−^, Cl^−^, and CO_3_^2−^. The introduction of NaNO_3_ and NaCl resulted in only minor decreases in SO_4_^2−^ uptake, with sorption capacities of 805.63 and 800.15 mg/g, respectively. These values correspond to retention of about 99.2% and 98.5% of the initial sorption capacity, indicating that the presence of NO_3_^−^ and Cl^−^ had a limited effect on SO_4_^2−^ sorption. A more pronounced decrease was observed in the presence of Na_2_CO_3_, for which the SO_4_^2−^ sorption capacity decreased to 766.25 mg/g, corresponding to about 94.3% retention relative to the control. The stronger influence of CO_3_^2−^ compared with the monovalent competing anions may be associated with its higher charge density and stronger interactions with the positively charged or ion-exchangeable sites involved in SO_4_^2−^ uptake. However, the relatively small changes observed in the presence of NO_3_^−^, Cl^−^, and CO_3_^2−^ demonstrate that the DN.XL3/Cu^2+^ sorbent maintains a high SO_4_^2−^ uptake under competitive conditions.

The results are particularly significant from the perspective of sequential pollutant removal and sorbent valorization. Conventional adsorption studies often regard a sorbent as exhausted once the primary contaminant has saturated its active sites. The present results demonstrate that after capturing Cu^2+^, the resulting Cu^2+^-loaded SN or DN cryogels remains highly active toward a second contaminant sulfate. Thus, the material can be viewed as undergoing a functional transformation rather than simple exhaustion. This concept provides what may be described as a “second life” for the sorbent.

#### 2.3.4. Mechanism of Cu^2+^ and SO_4_^2−^ Sorption

The kinetic and equilibrium results indicate that Cu^2+^ sorption onto DN cryogels is a heterogeneous, site-dependent process involving progressively occupied sorption sites and diffusion of Cu^2+^ ions toward less accessible sites within the polymer network. The better agreement of the experimental kinetic data with the pseudo-first-order model, together with the rapid initial uptake and subsequent approach to equilibrium, suggests that the sorption rate is predominantly associated with the availability and accessibility of sorption sites and mass-transfer processes rather than being controlled exclusively by chemisorption. At equilibrium, the superior fit of the Sips model indicates a heterogeneous distribution of Cu^2+^-binding sites with a finite saturation capacity.

**Figure 11:**
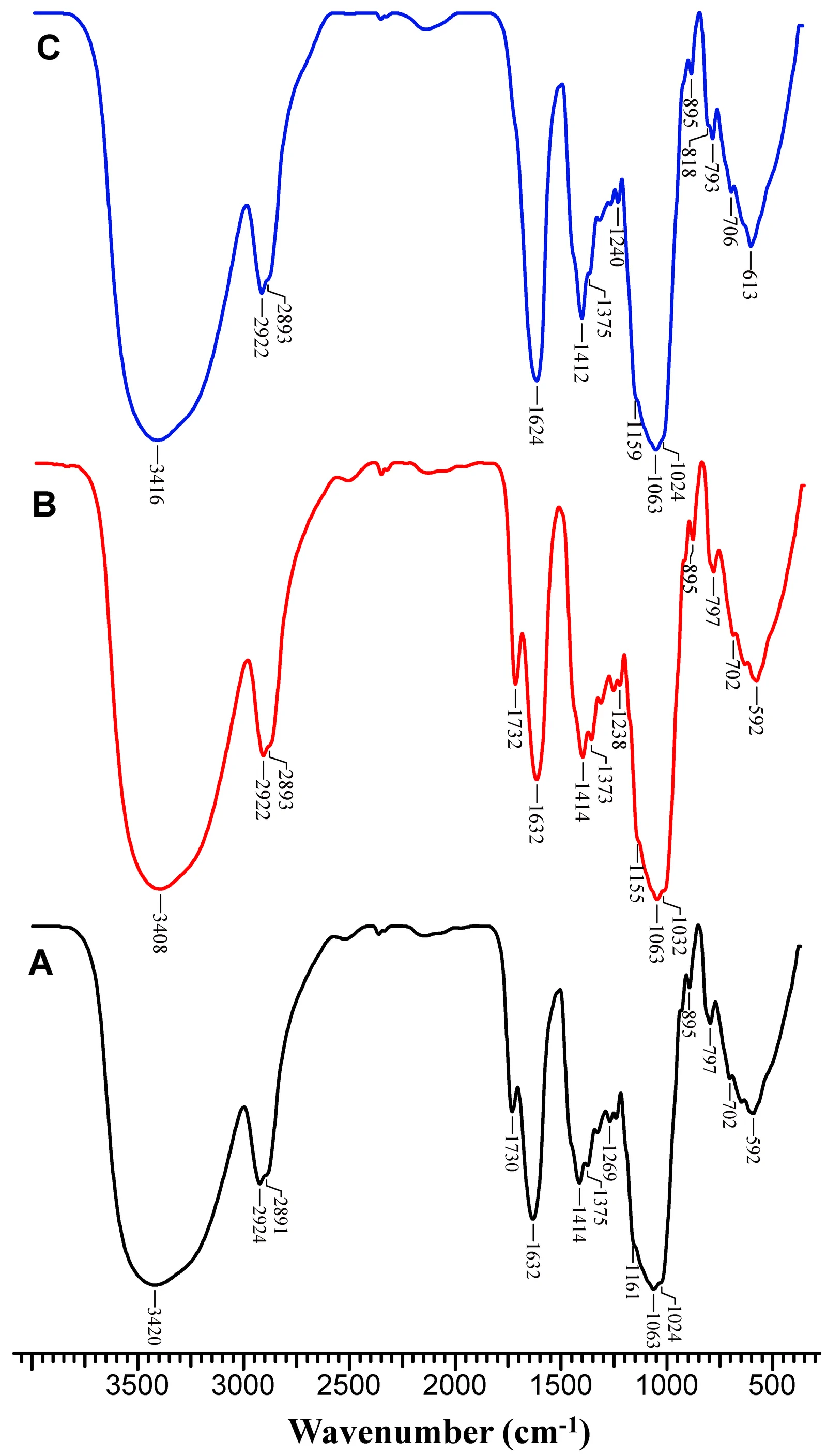
FT-IR spectra of DNXL3 cryogels after interaction with Cu^2+^ and SO_4_^2−^: (**A**) DN.XL3/Cu^2+^ composite; (**B**) DN.XL3/Cu^2+^ after the 5th cycle of Cu^2+^ sorption; and (**C**) DN.XL3/Cu^2+^/SO_4_^2−^ composite obtained at an initial SO_4_^2−^ concentration of 1000 mg/L.

FTIR studies (Figure 11) were performed to further understand the interaction mechanism of the functional groups from the DN.XL3 cryogels with Cu^2+^ and SO_4_^2−^ ions adsorbed from aqueous solutions.

Compared to the FTIR spectrum of the DN.XL3 cryogel before Cu^2+^ sorption (Figure 2), the FTIR spectra of the sorbent after the sorption of Cu^2+^ ions (sample DN.XL3/Cu^2+^) from aqueous solutions (Figure 11A) lead to:

(i) red shifts in the absorption bands associated with the –OH stretching vibrations from 3431 cm^−1^ for DN.XL3 cryogel to 3420 cm^−1^ for DN.XL3/Cu^2+^;
(ii) the absorption bands at 1740 cm^−1^ assigned to the carbonyl (C=O) stretching vibration of acetyl groups in XG spectrum (Figure S1) is red-shifted and visible at 1730 cm^−1^ for DN.XL3/Cu^2+^;
(iii) blue shifts in the asymmetric C=O stretching vibration of pyruvate groups, from 1614 cm^−1^ to 1632 cm^−1^;
(iv) down-shift and blue shifts in the band at 1410 cm^−1^ (DN.XL3, Figure 2) of the symmetric stretching vibration of carboxylate (-COO^−^) groups to 1414 cm^−1^ DN.XL3/Cu^2+^;
(v) down-shift and blue shifts in the deformation (bending) vibration of substituted benzene rings in LN, from 812 cm^−1^ (DN.XL3 cryogel) to 818 cm^−1^ (shoulder, DN.XL3/Cu^2+^).

All these findings are consistent with the previous results revealed for HMIs sorption onto polysaccharide-based sorbents [15,17,19,31,37]. Moreover, these results suggest the involvement of –OH, -C=O, and –COO^−^ functional groups from DN.XL3 cryogels in Cu^2+^ ions binding potentially through ion-exchange and/or chelation mechanism.

The FTIR spectrum of DN.XL3/Cu^2+^ after the fifth cycle of Cu^2+^ sorption was also recorded (Figure 11B). The presence of all characteristic absorption bands corresponding to XG and LN, together with shifts similar to those observed after the first Cu^2+^ sorption cycle indicates that the DN structure remains stable over repeated Cu^2+^ sorption cycles.

The FTIR spectrum of the Cu^2+^-loaded DN.XL3 composite after subsequent interaction with SO_4_^2−^ was also analyzed (Figure 11C). Several changes were observed compared with the spectrum of DN.XL3/Cu^2+^, including: (i) red shifts in the absorption band associated with the –OH stretching vibrations from 3420 cm^−1^ for DN.XL3/Cu^2+^ to 3416 cm^−1^ for DN.XL3/Cu^2+^/SO_4_^2−^; (ii) the disappearance of the absorption bands at 1740 cm^−1^, assigned to the C=O stretching vibration of acetyl groups; (iii) red shifts in the asymmetric C=O stretching vibration of the pyruvate groups, from 1632 cm^−1^ to 1624 cm^−1^; and (iv) downshifts of glycosidic skeletal vibrations, from 1159 cm^−1^, 1063 cm^−1^, and 1024 cm^−1^. Overall, these spectral changes indicate that the interaction of the Cu^2+^-loaded DN.XL3 composite with SO_4_^2−^ induces further modifications in the chemical environment of several functional groups within the cryogel, particularly those associated with -OH and C=O functionalities.

The high SO_4_^2−^ removal capacity observed for the DN.XL3 system can be associated with the Cu^2+^ ions previously sorbed onto the cryogel matrix. During Cu^2+^ sorption, the metal ions interact with the ionizable functional groups of the polysaccharide-based network, particularly –OH, –COO^−^, and C=O-containing groups, through ion-exchange and/or complexation mechanisms. The resulting Cu^2+^-loaded cryogel provides positively charged sites that can subsequently interact electrostatically with SO_4_^2−^ ions from aqueous solution. Thus, SO_4_^2−^ removal may occur through the attraction and retention of SO_4_^2−^ by the immobilized Cu^2+^ species, with the formation of copper sulfate within the cryogel matrix. This mechanism is consistent with the FTIR changes observed after interaction of the Cu^2+^-loaded DN.XL3 composite with SO_4_^2−^ (Figure 11C), which indicate changes in the chemical environment of several functional groups. Therefore, the high SO_4_^2−^ removal capacity of DN.XL3 may result from a sequential sorption process, in which Cu^2+^ is first immobilized by the cryogel and subsequently provides cationic sites capable of retaining sulfate ions. These results are in agreement with the previous results revealed for sulfate removal by various porous sorbents [83,84].

**Figure 12:**
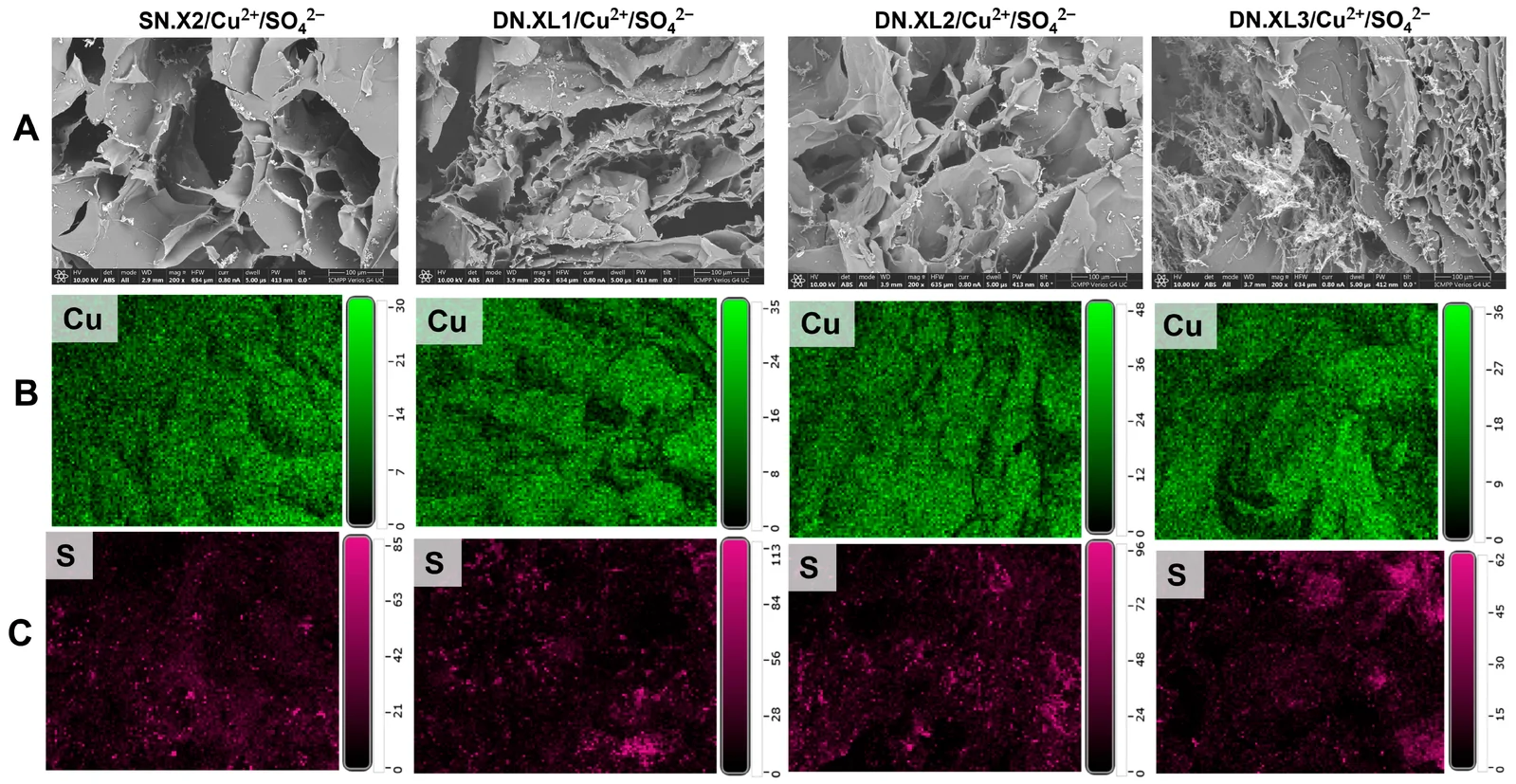
(**A**) SEM micrographs of SN/Cu^2+^/SO_4_^2−^ and DN/Cu^2+^/SO_4_^2−^ composites obtained at an initial SO_4_^2−^ concentration of 500 mg/L; (**B**) EDX mapping of SN/Cu^2+^/SO_4_^2−^ and DN/Cu^2+^/SO_4_^2−^ composites obtained at an initial SO_4_^2−^ concentration of 500 mg/L showing the presence of Cu on the surface of composites; (C) EDX mapping of SN/Cu^2+^/SO_4_^2−^ and DN/Cu^2+^/SO_4_^2−^ composites obtained at an initial SO_4_^2−^ concentration of 500 mg/L showing the presence of S on the surface of composites.

The internal structure of Cu^2+^-loaded SN and DN cryogels after interaction with SO_4_^2−^ was analyzed by SEM (Figure 12A; Figure S7A, Supporting Information). A macroporous morphology with interconnected pores was observed for all samples, together with the presence of small particles associated with the formation of CuSO_4_ on the surface of the cryogels. The EDX mapping of Cu^2+^-loaded SN and DN monoliths surface after interaction with SO_4_^2−^ shows the uniform distribution of Cu and S on the sorbent surface (Figure 12B,C; Figure S7B,C, Supporting Information).

The presence of Cu and S elements within the SN and DN matrices was further proved by EDX spectroscopy. EDX measurements were performed on the surface of SN/Cu^2+^/SO_4_^2−^ and DN/Cu^2+^/SO_4_^2−^ composites (Figures S8 and S9, Tables S2 and S3, Supporting Information). The elemental analysis of SN and DN monoliths surface after interaction with SO_4_^2−^ further sustains the high affinity of this sorbent for SO_4_^2−^ ions (Figures S8 and S9, Tables S2 and S3, Supporting Information).

## 3. Conclusions

This study demonstrated the potential of XG/LN DN cryogels as multifunctional sorbents for removal of various pollutants. The combination of XG and LN within an interconnected DN architecture produced materials with favorable physicochemical characteristics, high Cu^2+^ sorption capacity, substantial subsequent SO_4_^2−^ uptake, and antimicrobial activity. The SN XG-based cryogels exhibited experimental Cu^2+^ sorption capacities of 62.13–109.81 mg/g, whereas the DN cryogels achieved higher capacities of 111.87–120.84 mg/g. DN.XL3 exhibited the highest Cu^2+^ uptake, reaching 120.84 mg/g. The improved performance of the DN cryogels is attributed to the combined contribution of the chemical functionality of the biopolymer components (–OH, –COO^−^, and C=O functional groups) and the structural characteristics of the interconnected network, which can facilitate water penetration, ion transport, and accessibility of sorption domains.

Fitting of the equilibrium data by theoretical model isotherms demonstrated that the Sips equation provided the most appropriate description of Cu^2+^ sorption by the SN and DN cryogels. A particularly important finding was that the Cu^2+^-loaded networks retained significant functionality toward SO_4_^2−^. SO_4_^2−^ sorption capacities reached 694.53 mg/g for the SN.X2/Cu^2+^ composite and up to 812.53 mg/g for DN.XL3/Cu^2+^, with SO_4_^2−^ removal efficiencies reaching 81.3%. Thus, the initial Cu^2+^ sorption step does not simply exhaust the material; the Cu^2+^-loaded networks remained capable of subsequent SO_4_^2−^ capture. This behavior introduces the concept of a “second life” for the sorbent, in which the material can potentially be used sequentially for the removal of anionic contaminants.

In addition to their sorption performance, the developed cryogels demonstrated antimicrobial activity against *E. coli*, *S. typhimurium*, *L. monocytogenes*, and *S. aureus*, representing Gram-negative and Gram-positive bacterial species, respectively. This finding is particularly relevant for water-treatment applications because microbial contamination can coexist with inorganic pollutants in real water systems. The combination of antimicrobial activity with Cu^2+^ and SO_4_^2−^ removal therefore provides the cryogels with a broader functional profile than that of a conventional adsorbent.

## 4. Materials and Methods

### 4.1. Materials

XG, supplied as Satiaxane CX 910, was obtained from Degusa Texturant Systems (Saint-Germain-en-Laye, France) and employed as received, without any further purification. The viscosity-average molecular weight (M_v_) of the XG was 1980 kg/mol and was determined the procedure reported previously [85]. The degrees of acetylation and pyruvate substitution of the XG were determined by back-titration and according to the method developed by Sloneker and Orentas [85,86]. The analysis indicated that pyruvate groups were present on about one-third of the repeating saccharide units, whereas acetyl groups were associated with about two-thirds of the units. LN derived from annual plants was used as LN source and contains 2.43 mmol phenolic OH groups/g [32]. ECH, purum, ≥99% (Sigma-Aldrich, Darmstadt, Germany) was used as the cross-linking agent. NaOH, as pellets, was purchased from Chemical Company and was used to prepare NaOH solution of 2 M either to solubilized LN or as reaction medium for successful cross-linking of XG or LN by ECH.

### 4.2. Methods

#### 4.2.1. Preparation of SN Cryogels

The SN cryogels based on XG were prepared as monoliths by cross-linking XG with ECH in an alkaline medium at −18 °C. The parameter varied during the preparation of the SN cryogels was the concentration of the cross-linker. ECH was used as the cross-linking agent at molar ratios of ECH to XG ranging from 3:100 to 30:100 (Table 1). First, an XG solution with a concentration of 6 wt% was prepared. Subsequently, 5 g of the XG solution (6 wt%) was mixed with 1 mL of 2 M NaOH solution, followed by the addition of the corresponding amount of cross-linker under vigorous stirring (~600 rpm). The resulting reaction mixture was then transferred into 2 mL syringes, which were sealed with Parafilm and placed in a cooling-bath thermostat (CC1-K6 Huber Cryostat, Huber, Offenburg, Germany) maintained at −18 °C. After 24 h, the syringes were removed from the cryostat and allowed to thaw at room temperature (RT). Once completely thawed, the samples were removed from the syringes, cut into 1 cm pieces (monoliths), and transferred to Berzelius beakers. For purification, the XG-based cryogels were subjected to an extensive washing procedure with hot distilled water (40 °C) to remove unreacted ECH, soluble reaction products, and other low-molecular-weight species. The washing water was replaced every 30 min during the first 4 h and subsequently every 12 h for an additional 48 h. Finally, the samples were freeze-dried for 48 h at −57 °C and 0.045 mbar using a Freeze Dryer Biobase BK-FD10S (Jinan, China).

#### 4.2.2. Preparation of DN Cryogels

The DN cryogels were prepared using the SN.X2 cryogel obtained as described in Section 4.2.1. The SN.X2 cryogel was first swollen to equilibrium (approximately 1 h) in a 25 wt% LN solution prepared in 2 M NaOH. To stabilize the LN within the XG-based network, ECH was subsequently used as the cross-linking agent at molar ratios of ECH to phenolic OH groups ranging from 8:100 to 20:100 (Table 1). The samples were then placed in weighing bottles and transferred to a cooling-bath thermostat (CC1-K6 Huber Cryostat, Huber) maintained at −18 °C for 24 h. Afterward, the samples were removed from the cryostat and allowed to thaw at RT. Once completely thawed, the samples were removed from the weighing bottles, transferred to Berzelius beakers, and washed every 15 min. The water collected during the washing process was transferred to glass containers and analyzed by UV–Vis spectrophotometry to determine the amount of LN that was not incorporated into the XG-based cryogels. Following washing, the samples were freeze-dried for 48 h at −57 °C and 0.045 mbar using a Freeze Dryer Biobase BK-FD10S (Jinan, China).

#### 4.2.3. Gel Fraction Yield

The cross-linking efficiency of SN and DN cryogels was determined by estimation of the gel fraction yield (GFY, %), using Equation (4):

(4) $$\mathrm{GFY},\;\%\;=\frac{W_{d}}{\;W_{m}}\;\times 100$$

where *W_d_* (g) is the weight of lyophilized SN or DN cryogels and *W_m_* (g) is the weight of all materials involved in the preparation of cryogels. The GFY was reported as the average values of three repeated experiments.

#### 4.2.4. UV–Vis Analysis of Washing Solutions

The release of LN from DN cryogels during the washing process was quantified by UV–Vis spectrophotometry. A calibration curve was first established using alkali-soluble lignin. For this purpose, about 50 mg of LN was dissolved in a 50 mL volumetric flask using 2 M NaOH under ultrasonic agitation. After complete dissolution, the solution volume was adjusted to the mark with 2 M NaOH, yielding a stock solution with a LN concentration of 1 mg/mL. A series of seven standard solutions was prepared by transferring different aliquots from the stock solution into 5 mL volumetric flasks.

The absorbance of the standards was measured at 288 nm using a UV–Vis spectrophotometer (UV–vis SPECORD200, Carl Zeiss, Jena, Germany), and a calibration curve of absorbance vs. LN concentration was constructed. A linear relationship consistent with the Beer–Lambert law was obtained and subsequently used for the determination of LN concentrations in the washing solutions resulted during the purification step of DN cryogels.

#### 4.2.5. FT-IR Analysis

FT-IR spectroscopy was employed to confirm the formation of the SN and DN cryogels and to investigate the possible interactions of the matrix functional groups with Cu^2+^ and SO_4_^2−^ ions. The spectra were acquired using a Bruker Vertex FT-IR spectrometer (Bruker, Ettlingen, Germany) in the 4000–400 cm^−1^ wavenumber region with a spectral resolution of 2 cm^−1^. Analyses were performed using the KBr pellet technique, with approximately 5 mg of sample per pellet. Prior to FT-IR measurements, the cryogels were frozen in liquid nitrogen, then grounded, and finally dried under vacuum over P_2_O_5_.

#### 4.2.6. SEM Characterization

The internal morphology of the SN and DN cryogels before and after sorption of Cu^2+^ and SO_4_^2−^ ions was examined on fractured cross-sections using a Quanta 200 FEI environmental scanning electron microscope (ESEM) (FEI Company, Hillsboro, OR, USA) operated at 20 kV under low-vacuum conditions. The ESEM having coupled an energy-dispersive X-ray silicon-drift detector facilitated the rapid identification of elemental composition and the acquisition of corresponding elemental maps.

#### 4.2.7. Water Uptake Kinetics

The water uptake behavior of the SN and DN cryogels was investigated by immersing approximately 0.02 g of dried samples in 10 mL of distilled water at RT. At predetermined time intervals, the samples were removed from the medium, gently blotted with filter paper to eliminate the excess of water, and then weighed. The water uptake (*WU*, g/g) was determined using Equation (5):

(5) $$WU=\frac{W_{wet}-W_{dry}}{W_{dry}}$$

where *W_wet_* represents the weight of swollen SN or DN cryogels at time *t*, and *W_dry_* corresponds to the weight of the lyophilized cryogels. All experiments were performed in triplicate, and the reported values represent mean ± standard deviation.

#### 4.2.8. Dynamic Vapor Sorption (DVS)

The S_GAB_ and S_BET_ were assessed from DVS isotherms. The measurements were conducted at 25 °C using a fully automated IGAsorp gravimetric sorption analyzer (Hiden analytical, Warrington, UK). The relative humidity (RH) progressively increased from 0 to 90% in 10% increments. An equilibration period of 50–60 min was allowed at each RH level. Subsequently, the RH was reduced stepwise from 90 to 0%, using the same 10% increments, to obtain the desorption isotherms.

#### 4.2.9. Uniaxial Mechanical Tests

Mechanical properties were evaluated on fully swollen SN or DN cryogels at RT using a Shimadzu EZ-SX testing system (Kyoto, Japan). Cylindrical specimens with approximate dimensions of 8–10 mm in diameter and 7–9 mm in height were subjected to uniaxial compression at a crosshead speed of 1 mm/min under a maximum load of 50 N. Prior to testing, a preload of 0.1 N was applied to ensure proper contact between the sample surface and the parallel compression plates. Compressive stress (σ), strain (ε), and compressive elastic modulus were calculated following a previously reported methodology for similar porous composite materials [10,65].

#### 4.2.10. Sorption Experiments

##### Sorption of Cu^2+^ Ions

The adsorption capacity of Cu^2+^ ions from aqueous media was studied with respect to sorbent dose, contact time and initial metal concentration by batch procedure. To investigate the influence of sorbent dose on the sorption capacity, various amounts of either SN or DN cryogels (0.01–0.035 g) were added into 10 mL of Cu^2+^ solution (200 mg/L). The effect of contact time on the sorption capacity was investigated using 20 mg of sorbent. The initial Cu^2+^ concentration was set at 100 mg/L, and the initial solution pH was adjusted to 5.5. Contact times ranging from 15 to 480 min were investigated.

For equilibrium studies, the initial Cu^2+^ concentration was varied between 50 and 1000 mg/L. In a typical experiment, 20 mg of cryogel sorbent was added to 10 mL of Cu^2+^ ions solution, and the adsorption process was evaluated as a function of the initial metal ion concentration at room temperature. The contact time (8 h) and sorbent dosage were kept constant. The experiments were conducted at pH 5.5, a value previously reported as optimal for Cu^2+^ uptake in related systems [10,15]. After adsorption, the cryogel samples were separated by filtration, and the remaining Cu^2+^ concentration in solution was determined using flame atomic absorption spectrometry (FAAS) with a high-resolution ContrAA 300 instrument (Analytik, Jena, Germany) equipped with a xenon continuum radiation source. The aspiration rate was set to approximately 5 mL/min, and all measurements were performed in triplicate. The residual metal ion concentration was quantified at the characteristic wavelength of 324 nm for Cu^2+^. The equilibrium adsorption capacity (q_e_, mg/g) was calculated using Equation (6) [87]:

(6) $$q_{e}=\frac{(C_{0}-C_{e})V}{W}$$

where C_0_ and C_e_ are the initial and equilibrium concentrations of Cu^2+^ (mg/L), respectively, V is the solution volume (L), and W is the mass of sorbent (g).

The coefficient of determination (R^2^) together with the nonlinear chi-square (χ^2^) test Equation (7) was used to evaluate the fit of the isotherm models applied to the experimental data:

(7) $$\chi^{2}=\sum \frac{{\left(q_{e,\exp}-q_{e,cal}\right)}^{2}}{q_{e,cal}}$$

where q_e,exp_ and q_e,cal_ are the experimentally obtained and model-predicted adsorption capacities (mg/g), respectively.

##### Reusability Studies

The reusability of the SN and DN sorbents was assessed through consecutive sorption–desorption experiments. For the desorption tests, 0.02 g of Cu^2+^-loaded SN or DN cryogels was used in batch mode immediately following the sorption step. A 0.1 M HCl solution was employed as the desorbing agent in all experiments. After each desorption step, the recovered sorbents were reused in subsequent sorption–desorption cycles, for a total of five consecutive cycles.

##### Sorption of SO_4_^2−^ Ions

The sorption of sulfate ions was investigated using 10 mg of composite sorbent previously loaded with Cu^2+^ at two different initial concentrations (C_i_(Cu^2+^) = 500 and 1000 mg/L). The Cu^2+^-loaded materials were then kept in contact with 10 mL of SO_4_^2−^ solutions having initial concentrations of 400 and 1000 mg/L. The experiments were conducted at pH 6, with a contact time of 8 h at RT under constant conditions. The concentration of sulfate ions was determined using a turbidimetric method based on a calibration curve [88]. The method is based on the precipitation of sulfate ions with barium chloride (BaCl_2_), forming insoluble barium sulfate (BaSO_4_), which causes turbidity in the solution. The intensity of the resulting turbidity is proportional to the sulfate concentration and follows the principles of Lambert–Beer’s law. The measurements were performed at 490 nm, a wavelength selected from the UV–Vis spectrum of a standard sulfate solution after the addition of BaCl_2_. The absorbance values (turbidity signal) obtained for standard solutions of known concentration were used to construct the calibration curve, which was subsequently applied to quantify the sulfate ions in the samples.

##### Influence of Competing Ions on Sorption of Cu^2+^ Ions

To assess the effect of these competing ions on sorption capacity, 10 mL of an aqueous Cu^2+^ solution (200 mg/L, prepared in 0.1 M MgCl_2_ or 0.1 M CaCl_2_) was added to a flask containing about 0.02 g of dried DN cryogels. The mixtures were maintained at room temperature for 8 h. After sorption, the residual metal ion concentration was determined by AAS and the amount of Cu^2+^ adsorbed onto the DN cryogels was then calculated using Equation (6).

##### Selectivity for SO_4_^2−^ Ions

To evaluate the influence of coexisting anions on sulfate removal by the DN composite cryogels, sorption experiments were conducted in the presence of three competing anions: NO_3_^−^, Cl^−^, and CO_3_^2−^. The initial sulfate concentration was fixed at 1000 mg/L, the pH was adjusted to 6.0, and the sorbent dose was set at 20 mg. The experiments were performed at room temperature with a contact time of 8 h. Each competing anion was added at an initial concentration corresponding to a 1:1 molar ratio relative to SO_4_^2−^.

#### 4.2.11. Antibacterial Activity

Gram-negative bacterial strains, *Escherichia coli* (ATCC 25922) and *Salmonella Typhimurium* (ATCC 14028), and Gram-positive bacterial strains, *Staphylococcus aureus* (ATCC 25933) and *Listeria monocytogenes* (ATCC 7644), were used to evaluate the bacterial inhibition properties of the prepared cryogels. The experimental procedure was adapted from previously reported protocols [15,51,62]. Prior to testing, the cryogel samples were sterilized by autoclaving at 110 °C and 0.5 bar for 20 min. The sterilized samples were subsequently placed in sterile Petri dishes and inoculated with 1 mL of a standardized bacterial suspension corresponding to each ATCC strain. The samples were incubated in the dark at 25 °C for 24 h.

For comparison, 1 cm^2^ fragments of sterile wrapping paper were used as reference control samples and subjected to the same experimental procedure. Following incubation, 100 µL aliquots were collected from each Petri dish and spread onto the appropriate selective culture media: MacConkey agar for *E. coli* and *S. Typhimurium*, and Chapman agar for *S. aureus* and *L. monocytogenes*. The inoculated agar plates were subsequently incubated at 37 ± 1 °C for 24 h, after which the resulting colonies were counted.

The antibacterial activity was evaluated based on the reduction in recoverable viable bacterial colonies in the cryogel-containing samples relative to the corresponding control samples. The inhibition percentage was calculated using Equation (8):

(8) Inhibition (%) = [(N_0_ − N_s_)/N_0_] × 100

where N_0_ represents the number of viable colonies recovered from the control sample and N_s_ represents the number of viable colonies recovered following exposure to the SN or DN cryogel. A value of 100% therefore indicates that no viable colonies were detected in the analyzed aliquot under the experimental conditions, rather than providing direct evidence of complete bacterial eradication from the cryogel system.

All experiments followed the requirements of SR ISO 7218 [89] and SR ISO 11133 [90]. The results are reported as the mean of two independent measurements for each tested sample.

## Figures and Tables

**Table 1:** The samples code, feed composition, GFY, and percentage of LN removed from the DN cryogels.

Sample Code	1st Network		2nd Network		GFY, %	LN Leached from the DN Cryogels (wt.%)
	XG, wt.%	ECH:XG ^1^	LN, wt.%	ECH:OH ^2^		
SN.X1	6	3:100	-	-	79.40 ± 0.23	-
SN.X2	6	6:100	-	-	80.44 ± 0.77	-
SN.X3	6	10:100	-	-	79.96 ± 1.42	-
SN.X4	6	15:100	-	-	89.16 ± 0.51	-
SN.X5	6	20:100	-	-	91.24 ± 1.06	-
SN.X6	6	30:100	-	-	88.92 ± 3.08	-
DN.XL1	6	6:100	25	8:100	-	41.24 ± 2.26
DN.XL2	6	6:100	25	15:100	-	23.12 ± 0.94
DN.XL3	6	6:100	25	20:100	-	9.10 ± 1.72

^1^ The ratio between ECH and XG was calculated as moles of ECH to 100 moles of repeating pentasaccharide units from XG. ^2^ The ratio between ECH and LN was determined as moles of ECH to 100 moles of phenolic OH (LN from annual plants contains 2.43 mmol phenolic OH groups/g) [32].

**Table 2:** Equilibrium isotherm parameters for Cu^2+^ sorption onto SNs.

Isotherm Model	Parameters	SN.X1	SN.X2	SN.X3	SN.X4	SN.X5	SN.X6
	q_m,exp_, mg/g	109.810	92.740	82.540	74.530	69.180	62.130
Langmuir	q_m_, mg/g	114.565	99.061	90.022	81.793	79.716	75.584
	K_L_, L/mg	0.069	0.041	0.029	0.025	0.015	0.010
	R ^2^	0.992	0.987	0.983	0.978	0.953	0.866
	χ ^2^	14.708	15.959	16.806	17.378	34.039	89.230
Freundlich	K_F_, L/mg	36.579	27.240	21.525	18.809	12.935	8.940
	1/n	0.183	0.201	0.218	0.222	0.268	0.308
	R ^2^	0.858	0.858	0.861	0.859	0.829	0.737
	χ ^2^	253.833	177.637	137.293	112.539	123.810	174.462
Sips	qm, mg/g	110.953	93.537	84.587	76.246	69.911	61.654
	a_s_	0.041	0.012	0.007	0.005	3.096 × 10^−4^	2.844 × 10^−7^
	1/n	0.813	0.696	0.702	0.672	0.498	0.284
	R ^2^	0.996	1.000	0.995	0.992	0.998	0.999
	χ ^2^	7.721	0.276	5.300	6.120	1.381	0.740

**Table 3:** Equilibrium isotherm parameters for Cu^2+^ sorption onto DNs.

Isotherm Model	Parameters	DN.XL1	DN.XL2	DN.XL3
Langmuir	q_m__,exp_, mg/g	111.870	114.730	120.840
	*q_m_*, mg/g	128.280	130.710	135.439
	*K_L_*, L/mg	0.015	0.017	0.020
	*R* ^2^	0.956	0.958	0.962
	*χ* ^2^	73.545	73.307	72.875
Freundlich	*K_F_*, L/mg	22.627	24.960	29.337
	1/*n*	0.255	0.244	0.227
	*R* ^2^	0.838	0.838	0.840
	*χ* ^2^	268.622	281.347	303.459
Sips	*qm*, mg/g	113.755	117.270	123.258
	*a_s_*	3.985 × 10^−4^	6.426 × 10^−4^	0.001
	1/*n*	0.519	0.543	0.570
	*R* ^2^	0.994	0.992	0.991
	*χ* ^2^	9.715	14.358	17.695

**Table 4:** Sorption of SO_4_^2−^ by SN/Cu^2+^ and DN/Cu^2+^ composites.

C_i_ Cu^2+^ mg/L	SN.X2		DN.XL1		DN.XL2		DN.XL3	
	C_i_(SO_4_^2−^), mg/L		C_i_(SO_4_^2−^), mg/L		C_i_(SO_4_^2−^), mg/L		C_i_(SO_4_^2−^), mg/L	
	400	1000	400	1000	400	1000	400	1000
	q_e_, mg/g	q_e_, mg/g	q_e_, mg/g	q_e_, mg/g	q_e_, mg/g	q_e_, mg/g	q_e_, mg/g	q_e_, mg/g
500	172.43	508.96	216.92	594.36	243.84	617.48	258.43	635.29
1000	201.84	694.53	247.83	751.83	285.76	796.54	319.82	812.53

## Data Availability

Data are contained within the article and Supplementary Materials.

## Supplementary Materials

The following supporting information can be downloaded at https://www.mdpi.com/article/10.3390/gels12100915/s1, Figure S1. FTIR spectrum of uncross-linked XG; Figure S2. SEM micrographs of the SN.X2 cryogels at different magnifications: (A) 150x; (B) 250x; Figure S3. Sorption-desorption isotherms of the XG-based cryogels; Figure S4. Representative photographs showing the antimicrobial effects of the tested samples against *Listeria monocytogenes*, *Escherichia coli*, and *Salmonella Typhimurium* after 24 h of incubation. For each organism, the control represents bacterial growth in the absence of the tested sample, while SN.X2, DN.XL1, DN.XL2, and DN.XL3 represent the corresponding treatments. The reduction in the number of visible colonies relative to the control indicates inhibition of bacterial growth. The figure illustrates the differential susceptibility of the three bacterial species to the tested samples, with progressively fewer colonies generally observed in treated plates compared with their respective controls; Figure S5. (A) Influence of sorbent dose on the sorption capacity (q_e_, mg/g) and removal efficiency (RE,%) of Cu^2+^ ions for SN.X2 cryogels; (B) Influence of contact time on the sorption capacity of Cu^2+^ ions for SN.X2 cryogels; Figure S6. (A) Influence of sorbent dose on the sorption capacity (q_e_, mg/g) and removal efficiency (RE,%) of Cu^2+^ ions for DN.XL3 cryogels; (B) Influence of contact time on the sorption capacity of Cu^2+^ ions for DN.XL3 cryogels; Figure S7. (A) SEM micrographs of SN/Cu^2+^/SO_4_^2−^ and DN/Cu^2+^/SO_4_^2−^ composites obtained at an initial SO_4_^2−^ concentration of 1000 mg/L; (B) EDX mapping of SN/Cu^2+^/SO_4_^2−^ and DN/Cu^2+^/SO_4_^2−^ composites obtained at an initial SO_4_^2−^ concentration of 1000 mg/L showing the presence of Cu on the surface of composites; (C) EDX mapping of SN/Cu^2+^/SO_4_^2−^ and DN/Cu^2+^/SO_4_^2−^ composites obtained at an initial SO_4_^2−^ concentration of 1000 mg/L showing the presence of S on the surface of composites; Figure S8. EDX profiles of SN/Cu^2+^/SO_4_^2−^ and DN/Cu^2+^/SO_4_^2−^ composites obtained at an initial SO_4_^2−^ concentration of 500 mg/L; Figure S9. EDX profiles of SN/Cu^2+^/SO_4_^2−^ and DN/Cu^2+^/SO_4_^2−^ composites obtained at an initial SO_4_^2−^ concentration of 1000 mg/L; Table S1. Kinetic parameters for the sorption of Cu^2+^ onto the XG-based cryogels; Table S2. Atomic percentages (%) of elements present on the surface of the SN and DN cryogels after interaction with SO_4_^2−^ (C_i_ = 500 mg/L); Table S3. Atomic percentages (%) of elements present on the surface of the SN and DN cryogels after interaction with SO_4_^2−^ (C_i_ = 1000 mg/L).

## References

[1] Fernández-Luqueño F., López-Valdez F., Gamero-Melo P., Luna-Suárez S., Aguilera-González E.N., Martínez A.I., del Socorro García-Guillermo M., Hernández-Martínez G., Herrera-Mendoza R., Álvarez-Garza M.A. (2013). Heavy metal pollution in drinking water-a global risk for human health: A review. Afr. J. Environ. Sci. Technol..

[2] Rehman M., Liu L., Wang Q., Saleem M.H., Bashir S., Ullah S., Peng D. (2019). Copper environmental toxicology, recent advances, and future outlook: A Review. Environ. Sci. Pollut. Res..

[3] Dippong T., Resz M.A. (2024). Chemical assessment of drinking water quality and associated human health risk of heavy metals in Gutai Mountains, Romania. Toxics.

[4] Stanescu S.G., Banica A.L., Dulama I.D., Stirbescu R.M., Coman M.D., Radulescu C. (2024). Assessment of drinking water quality and associated health risk: A statistical approach based on physicochemical parameters and heavy metals content. J. Sci. Arts.

[5] Blades B., Ayton S., Hung Y.H., Bush A.I., La Fontaine S. (2021). Copper and lipid metabolism: A reciprocal relationship. Biochim. Biophys. Acta Gen. Subj..

[6] Saravanan A., Senthil Kumar P., Jeevanantham S., Karishma S., Tajsabreen B., Yaashikaa P.R., Reshma B. (2021). Effective water/wastewater treatment methodologies for toxic pollutants removal: Processes and applications towards sustainable development. Chemosphere.

[7] Dhokpande S.R., Deshmukh S.M., Khandekar A., Sankhe A. (2024). A review outlook on methods for removal of heavy metal ions from wastewater. Sep. Purif. Technol..

[8] Jannat U.T., Haque M.A., Aumi S.A., Ray S.K., Moniruzzaman M., Jahan M.S., Shaikh M.A.A., Ehsan M.Q. (2026). Waste-derived-lignin based hydrogel as a reusable adsorbent for efficient removal of dyes from aqueous solution. RSC Adv..

[9] Alcalde-Garcia F., Prasher S., Kaliaguine S., Tavares J.R., Dumont M.-J. (2023). Desorption strategies and reusability of biopolymeric adsorbents and semisynthetic derivatives in hydrogel and hydrogel composites used in adsorption processes. ACS Eng. AU.

[10] Dinu M.V., Humelnicu D., Lazar M.M., Dragan E.S. (2025). Cationic composite cryogels as potential filters for removal of metallic pollutants, anionic dyes, and bacteria from water. ACS Appl. Eng. Mater..

[11] Alsaka L., Alsaka L., Altaee A., Zaidi S.J., Zhou J., Kazwini T. (2025). A review of hydrogel application in wastewater purification. Separations.

[12] Sandu T., Chiriac A.-L., Zaharia A., Iordache T.-V., Sarbu A. (2025). New trends in preparation and use of hydrogels for water treatment. Gels.

[13] Ahmaruzzaman M., Roy P., Bonilla-Petriciolet A., Badawi M., Ganachari S.V., Shetti P.N., Aminabhavi T.M. (2023). Polymeric hydrogels-based materials for wastewater treatment. Chemosphere.

[14] Sharma G., Verma Y., Dhiman P., Wang T., Jain S., García-Penas A., Lai C.W. (2026). Gellan gum–alginate/PVA-co-PAM crosslinked hydrogel for phenol adsorption: Fabrication, characterization, and performance. Int. J. Biol. Macromol..

[15] Ghiorghita C.A., Humelnicu D., Dinu M.V., Ignat M., Bonardd S., Díaz D.D., Dragan E.S. (2023). Polyelectrolyte complex composite cryogels with self-antibacterial properties and wide window for simultaneous removal of multiple contaminants. Chem. Eng. J..

[16] Verma Y., Zhan H., Sharma J., Verma A., Du H., Jindal Y., Patyal S., Sharma G., Dhiman P., Li L. (2026). Lignocellulose-based hydrogels for environmental remediation: Structure-function relationships, adsorption mechanism, and pathways towards sustainable water treatment. Carbohydr. Polym. Technol. Appl..

[17] Li C., Shen J., Wang J., Bao C., Li B., Liu L., Wang H., Zhang X. (2023). Highly compressible and macro-porous hydrogels via the synergy of cryogelation and double-network for efficient removal of Cr(VI). Int. J. Biol. Macromol..

[18] Savina I.N., Otero-Gonzalez L., Berillo D., Mohanan P.V., Kappalli S. (2023). Macroporous cryogel-based systems for water treatment applications and safety: Nanocomposite-based cryogels and bacteria-based bioreactors. Biomedical Applications and Toxicity of Nanomaterials.

[19] Amonkol P., Choochottiros C. (2026). Comparative study between cryogel and hydrogel-based chitosan: Relationship between structure, swelling ability, and Cu(II) adsorption ability. ACS Omega.

[20] Rogers Z.J., Bencherif S.A. (2019). Cryogelation and cryogels. Gels.

[21] He Y., Wang C., Wang C., Xiao Y., Lin W. (2021). An overview on collagen and gelatin-based cryogels: Fabrication, classification, properties and biomedical applications. Polymers.

[22] Baimenov A., Berillo D.A., Poulopoulos S.G., Inglezakis V.J. (2020). A review of cryogels synthesis, characterization and applications on the removal of heavy metals from aqueous solutions. Adv. Colloid Interface Sci..

[23] Satchanska G., Davidova S., Petrov P.D. (2024). Natural and synthetic polymers for biomedical and environmental applications. Polymers.

[24] Thakur S., Govender P.P., Mamo M.A., Tamulevicius S., Mishra Y.K., Thakur V.K. (2017). Progress in lignin hydrogels and nanocomposites for water purification: Future perspectives. Vacuum.

[25] Mohammed A.S.A., Naveed M., Jost N. (2021). Polysaccharides; Classification, Chemical Properties, and Future Perspective Applications in Fields of Pharmacology and Biological Medicine (A Review of Current Applications and Upcoming Potentialities). J. Polym. Environ..

[26] Qin L., Chen S., Xie L., Yu Q., Chen Y., Xie J. (2022). Recent advances in Mung bean polysaccharides: Extraction, physicochemical properties and biological activities. Process Biochem..

[27] Li H., Xing R., Wang Z., Li G. (2025). Advancements in xanthan gum-based film and coating for food packaging. Carbohydr. Polym..

[28] Raschip I.E., Paduraru-Mocanu O.M., Nita L.E., Dinu M.V. (2020). Antibacterial porous xanthan-based films containing flavoring agents evaluated by near infrared chemical imaging technique. J. Appl. Polym. Sci..

[29] Raschip I.E., Fifere N., Lazar M.M., Hitruc G.E., Dinu M.V. (2023). Ice-templated and cross-linked xanthan-based hydrogels: Towards tailor-made properties. Gels.

[30] Sharma G., Kumar A., Ghfar A.A., García-Peñas A., Naushad M., Stadler F.J. (2022). Fabrication and characterization of xanthan gum-cl-poly(Acrylamide-co-alginic acid) hydrogel for adsorption of cadmium ions from aqueous medium. Gels.

[31] Kashaudhan K., Pande P.P., Sharma J., Chaurasiya A., Shankar R., Nath A. (2025). Facile synthesis of XGX-based hydrogel and its application for the removal of heavy metal ions from aqueous solutions. J. Porous Mater..

[32] Raschip I.E., Hitruc G.E., Vasile C., Popescu M.C. (2013). Effect of the lignin type on the morphology and thermal properties of the xanthan/lignin hydrogels. Int. J. Biol. Macromol..

[33] Ge Y., Li Z. (2018). Application of lignin and its derivatives in adsorption of heavy metal ions in water: A Review. ACS Sustain. Chem. Eng..

[34] Sheraz M., Sun X.F., Siddiqui A., Hu S., Song Z. (2025). Research advances in natural polymers for environmental remediation. Polymers.

[35] Dragan E.S. (2014). Design and applications of interpenetrating polymer network hydrogels. A review. Chem. Eng. J..

[36] Dragan E.S., Humelnicu D., Dinu M.V. (2019). Development of chitosan-poly(ethyleneimine) based double network cryogels and their application as superadsorbents for phosphate. Carbohydr. Polym..

[37] Tang S., Yang J., Lin L., Peng K., Chen Y., Jin S., Yao W. (2020). Construction of physically crosslinked chitosan/sodium alginate/calcium ion double-network hydrogel and its application to heavy metal ions removal. Chem. Eng. J..

[38] Almeida A.P.C., Saraiva J.N., Cavaco G., Portela R.P., Leal C.R., Sobral R.G., Almeida P.L. (2022). Crosslinked bacterial cellulose hydrogels for biomedical applications. Eur. Polym. J..

[39] Schio R.R., Gonçalves J.O., Mallmann E.S., Pinto D., Dotto G.L. (2021). Development of a biosponge based on *Luffa cylindrica* and crosslinked chitosan for Allura red AC adsorption. Int. J. Biol. Macromol..

[40] Pawar A.Y., Jadhav S.R., Derle D.V. (2021). Fabrication of chitosan based antibacterial implant for multibacterial bone infection. J. Pharm. Res. Int..

[41] Dyatlov V., Seregina T., Luss A., Zaitsev V., Artyukhov A., Shtilman M., Chumakova A., Kushnerev K., Tsatsakis A., Mezhuev Y. (2020). Immobilization of amikacin on dextran: Biocomposite materials that release an antibiotic in the presence of bacterial dextranase. Polym. Int..

[42] Mirtaghavi A., Baldwin A., Tanideh N., Zarei M., Muthuraj R., Cao Y., Zhao G., Geng J., Jin H., Luo J. (2020). Crosslinked porous three-dimensional cellulose nanofibers-gelatine biocomposite scaffolds for tissue regeneration. Int. J. Biol. Macromol..

[43] Bakravi A., Ahamadian Y., Hashemi H., Namazi H. (2018). Synthesis of gelatin-based biodegradable hydrogel nanocomposite and their application as drug delivery agent. Adv. Polym. Technol..

[44] Asnani G.P., Kokare C.R. (2018). In vitro and in vivo evaluation of colon cancer targeted epichlorohydrin crosslinked Portulaca-alginate beads. Bio. Mol. Concepts.

[45] Islam M.S., Alam M.N., van de Ven T.G.M. (2020). Sustainable cellulose-based hydrogel for dewatering of orange juice. Cellulose.

[46] Xie Y., Pan Y., Cai P. (2022). Cellulose-based antimicrobial films incroporated with ZnO nanopillars on surface as biodegradable and antimicrobial packaging. Food Chem..

[47] Meng X., Dong Y., Zhao Y., Liang L. (2020). Preparation and modification of cellulose sponge and application of oil/water separation. RSC Adv..

[48] Romano K.P., Newman A.G., Zahran R.W., Millard J.T. (2007). DNA interstrand cross-linking by epichlorohydrin. Chem. Res. Toxicol..

[49] Yang Q.Z., Fan C.J., Yang X.G., Liao L.Q., Liu L.J. (2016). Facile synthesis of biocompatible polyglycerol hydrogel based on epichlorohydrin. J. Appl. Polym. Sci..

[50] Wei X., Yi W., Chen W., Ma X., Lau W.B., Wang H., Yi D. (2010). Clinical outcomes with the epicholorohydrin-modified porcine aortic heart valve: A 15-year follow-up. Ann. Thorac. Surg..

[51] Raschip I.E., Platon I.-V., Fifere N., Darie-Nita R.-N., Aprotosoaie A.C., Dinu M.V. (2025). Stabilization of anthocyanins in xanthan based systems for synergistic cryogels with enhanced physicochemical and biological properties for visual freshness monitoring of Prussian carp (Carassius gibelio). Food Hydrocoll..

[52] Zheng M., Lian F., Xiong Y., Liu B., Zhu Y., Miao S., Zhang L., Zheng B. (2019). The synthesis and characterization of a xanthan gum-acrylamide trimethylolpropane triglycidyl ether hydrogel. Food Chem..

[53] Kang M., Oderinde O., Liu S., Huang Q., Ma W., Yao F., Fu G. (2019). Characterization of Xanthan gum-based hydrogel with Fe^3+^ ions coordination and its reversible sol-gel conversion. Carbohydr. Polym..

[54] Zhang Q., Hu X.M., Wu M.Y., Wang M.M., Zhao Y.Y., Li T.T. (2019). Synthesis and performance characterization of poly(vinylalcohol)-xanthan gum composite hydrogel. React. Funct. Polym..

[55] Brunchi C.-E., Bercea M., Morariu S., Avadanei M. (2016). Investigations on the interactions between xanthan gum and poly(vinylalcohol) in solid state and aqueous solutions. Eur. Polym. J..

[56] Elella M.H.A., Sabaa M.W., El Hafeez E.A., Mohamed R.R. (2019). Crystal violet dye removal using cross-linked grafted xanthan gum. Int. J. Biol. Macromol..

[57] Boeriu C.G., Bravo D., Gosselink R.J.A., van Dam J.E.G. (2004). Characterisation of structure-dependent functional properties of lignin with infrared spectroscopy. Ind. Crops Prod..

[58] Lv P., Almeida G., Perré P. (2015). TGA-FTIR analysis of torrefaction of lignocellulosic components (cellulose, xylan, lignin) in isothermal conditions over a wide range of time durations. BioResources.

[59] Joukhdar H., Seifert A., Jüngst T., Groll J., Lord M.S., Rnjak-Kovacina J. (2021). Ice templating soft matter: Fundamental principles and fabrication approaches to tailor pore structure and morphology and their biomedical applications. Adv. Mater..

[60] Lozinsky V.I., Okay O., Okay O. (2014). Basic principles of cryotropic gelation. Polymeric Cryogels, Macroporous Gels with Remarkable Properties.

[61] Okay O., Lozinsky V.I., Okay O. (2014). Synthesis, structure-property relationships of cryogels. Polymeric Cryogels, Macroporous Gels with Remarkable Properties.

[62] Platon I.-V., Ghiorghita C.-A., Lazar M.M., Raschip I.E., Dinu M.V. (2023). Chitosan sponges with instantaneous shape recovery and multistrain antibacterial activity for controlled release of plant-derived polyphenols. Int. J. Mol. Sci..

[63] Ak F., Oztoprak Z., Karakutuk I., Okay O. (2013). Macroporous silk fibroin cryogels. Biomacromolecules.

[64] Zaharia C., Tudora M.R., Stancu I.C., Galateanu B., Lungu A., Cincu C. (2012). Characterization and deposition behavior of silk hydrogels soaked in simulated body fluid. Mater. Sci. Eng. C.

[65] Dragan E.S., Dinu M.V., Ghiorghita C.A. (2022). Chitosan-based polyelectrolyte complex cryogels with elasticity, toughness and delivery of curcumin engineered by polyions pair and cryostructuration steps. Gels.

[66] Zhao Q., Sun J., Wu X., Lin Y. (2011). Macroporous double-network cryogels: Formation mechanism, enhanced mechanical strength and temperature/pH dual sensitivity. Soft Matter.

[67] Köhnkea T., Elder T., Theliander H., Ragauskas A.J. (2014). Ice templated and cross-linked xylan/nanocrystalline cellulose hydrogels. Carbohydr. Polym..

[68] Maharjan B., Park J., Kaliannagounder V.K., Awasthi G.P., Joshi M.K., Park C.H., Kim C.S. (2020). Regenerated cellulose nanofiber reinforced chitosan hydrogel scaffolds for bone tissue engineering. Carbohydr. Polym..

[69] Wei W., Hu X., Qi X., Yu H., Liu Y., Li J., Zhang J., Dong W. (2015). A novel thermo-responsive hydrogel based on salecan and poly(N-isopropylacrylamide): Synthesis and characterization. Colloids Surf. B Biointerfaces.

[70] Boarino A., Klok H.-A. (2023). Opportunities and challenges for lignin valorization in food packaging, antimicrobial, and agricultural applications. Biomacromolecules.

[71] Bouarab-Chibane L., Forquet V., Lantéri P., Clément Y., Léonard-Akkari L., Oulahal N., Degraeve P., Bordes C. (2019). Antibacterial properties of polyphenols: Characterization and QSAR (Quantitative Structure−Activity Relationship) models. Front. Microbiol..

[72] Miklasinska-Majdanik M., Kępa M., Wojtyczka R., Idzik D., Wąsik T. (2018). Phenolic compounds diminish antibiotic resistance of *Staphylococcus Aureus* Clinical Strains. Int. J. Environ. Res. Public Health.

[73] Lourençon T.V., de Lima G.G., Ribeiro C.S.P., Hansel F.A., Maciel G.M., da Silva K., Winnischofer S.M.B., de Muniz G.I.B., Magalhães W.L.E. (2021). Antioxidant, antibacterial and antitumoural activities of kraft lignin from hardwood fractionated by acid precipitation. Int. J. Biol. Macromol..

[74] Dong X., Dong M., Lu Y., Turley A., Jin T., Wu C. (2011). Antimicrobial and antioxidant activities of lignin from residue of corn stover to ethanol production. Ind. Crops Prod..

[75] Richter A.P., Brown J.S., Bharti B., Wang A., Gangwal S., Houck K., Cohen Hubal E.A., Paunov V.N., Stoyanov S.D., Velev O.D. (2015). An environmentally benign antimicrobial nanoparticle based on a silver-infused lignin core. Nat. Nanotechnol..

[76] El-Nemr K.F., Mohamed H.R., Ali M.A., Fathy R.M., Dhmees A.S. (2020). Polyvinyl alcohol/gelatin irradiated blends filled by lignin as green filler for antimicrobial packaging materials. Int. J. Environ. Anal. Chem..

[77] Alzagameem A., Klein S.E., Bergs M., Do X.T., Korte I., Dohlen S., Hüwe C., Kreyenschmidt J., Kamm B., Larkins M. (2019). Antimicrobial activity of lignin and lignin-derived cellulose and chitosan composites against selected pathogenic and spoilage microorganisms. Polymers.

[78] Lee E., Song Y., Lee S. (2019). Crosslinking of lignin/poly(vinyl alcohol) nanocomposite fiber webs and their antimicrobial and ultraviolet-protective properties. Text. Res. J..

[79] Al-Ghouti M.A., Da’ana D.A. (2020). Guidelines for the use and interpretation of adsorption isotherm models: A review. J. Hazard. Mater..

[80] Langmuir I. (1916). The constitution and fundamental properties of solids and liquids. Part I Solids J. Am. Chem. Soc..

[81] Kundu S., Gupta A.K. (2006). Arsenic adsorption onto iron oxide-coated cement (IOCC): Regression analysis of equilibrium data with several isotherm models and their optimization. Chem. Eng. J..

[82] Saadi R., Saadi Z., Fazaeli R., Fard N.E. (2015). Monolayer and multilayer adsorption isotherm models for sorption from aqueous media. Korean J. Chem. Eng..

[83] Sadeghalvad B., Khorshidi N., Azadmehr A., Sillanpaa M. (2021). Sorption, mechanism, and behavior of sulfate on various adsorbents: A critical review. Chemosphere.

[84] Steiger B.G.K., Solgi M., Wilson L.D. (2025). Biopolymers to composite adsorbents for sulfate removal: From conventional to sustainable systems. Adv. Colloid Interface Sci..

[85] Pelletier E., Viebke C., Meadows J., Williams P.A. (2011). Rheological study of the order–disorder conformational transition of xanthan gum. Biopolymers.

[86] Sloneker J.H., Orentas D.G. (1962). Pyruvic acid, a unique component of an exocellular bacterial polysaccharide. Nature.

[87] Dinu M.V., Dinu I.A., Lazar M.M., Dragan E.S. (2018). Insights into the mechanism of Cu^2+^ binding onto chitosan based cryogel composites: Equilibrium, kinetics and thermodynamics studies. Cellul. Chem. Technol..

[88] Anechitei L., Cojocaru T., Munteanu I.G., Bulgariu L. (2019). Simple methods for quantitative determination of sulphate ions from aqueous media with industrial applications. Bull. Inst. Pol. Iasi.

[89] (2024). Microbiology of the Food Chain—General Requirements and Guidance for Microbiological Examinations SR.

[90] (2014). Microbiology of Food, Animal Feed and Water—Preparation, Production, Storage and Performance Testing of Culture Media.

