# Supplementary material for: Xanthan Gum/Lignin Double-Interpenetrating Network Cryogels: Preparation, Characterization, and Water Purification Performance
Source: Gels. 2026 Oct 11;12(10):915. doi: 10.3390/gels12100915 (PMC13704480; doi:10.3390/gels12100915)
Supplement: Supplementary file 1 [file gels-12-00915-s001.zip › gels-4576307-supplementary.pdf]

Supplementary Information

Xanthan Gum/Lignin Double-Interpenetrating Network Cryogels: Preparation, Characterization, and Water Purification Performance

Maria Marinela Lazar [1,*] Doina Humelnicu[2], Irina Elena Raschip[1], Raluca-Elena Avram[1], and Maria Valentina Dinu[1]

[1] Department of Functional Polymers, "Petru Poni" Institute of Macromolecular Chemistry, Grigore Ghica Voda Alley 41A, 700487 Iasi, Romania; iecoj@icmpp.ro (I.E.R.); munteanu.raluca@icmpp.ro (R.-E.A.); vdinu@icmpp.ro (M.V.D.);

[2] Faculty of Chemistry, Alexandru Ioan Cuza University of Iasi, Carol I Bd. 11, 700506 Iasi, Romania; doinah@uaic.ro (D.H.)

* Correspondence: maria.lazar@icmpp.ro

Figure S1. FTIR spectrum of uncross-linked XG.

Figure S2. SEM micrographs of the SN.X2 cryogels at different magnifications: (A) 150x; (B) 250x.

Figure S3. Sorption-desorption isotherms of the XG-based cryogels.

Figure S4. Representative photographs showing the antimicrobial effects of the tested samples against *Listeria monocytogenes*, *Escherichia coli*, and *Salmonella Typhimurium* after 24 h of incubation. For each organism, the control represents bacterial growth in the absence of the tested sample, while SN.X2, DN.XL1, DN.XL2, and DN.XL3 represent the corresponding treatments. The reduction in the number of visible colonies relative to the control indicates inhibition of bacterial growth. The figure illustrates the differential susceptibility of the three bacterial species to the tested samples, with progressively fewer colonies generally observed in treated plates compared with their respective controls.

Figure S5. (A) Influence of sorbent dose on the sorption capacity (q_e, mg/g) and removal efficiency (RE,%) of Cu^{2+} ions for SN.X2 cryogels; (B) Influence of contact time on the sorption capacity of Cu^{2+} ions for SN.X2 cryogels.

Figure S6. (A) Influence of sorbent dose on the sorption capacity (q_e, mg/g) and removal efficiency (RE,%) of Cu^{2+} ions for DN.XL3 cryogels; (B) Influence of contact time on the sorption capacity of Cu^{2+} ions for DN.XL3 cryogels.

Figure S7. (A) SEM micrographs of SN/Cu^{2+}/ SO_4^{2-} and DN/ Cu^{2+}/ SO_4^{2-} composites obtained at an initial SO_4^{2-} concentration of 1000 mg/L; (B) EDX mapping of SN/Cu^{2+}/ SO_4^{2-} and DN/ Cu^{2+}/ SO_4^{2-} composites obtained at an initial SO_4^{2-} concentration of 1000 mg/L showing the presence of Cu on the surface of composites; (C) EDX mapping of SN/Cu^{2+}/ SO_4^{2-} and DN/ Cu^{2+}/ SO_4^{2-} composites obtained at an initial SO_4^{2-} concentration of 1000 mg/L showing the presence of S on the surface of composites.

Figure S8. EDX profiles of SN/Cu^{2+}/ SO_4^{2-} and DN/ Cu^{2+}/ SO_4^{2-} composites obtained at an initial SO_4^{2-} concentration of 500 mg/L.

Figure S9. EDX profiles of SN/Cu^{2+}/ SO_4^{2-} and DN/ Cu^{2+}/ SO_4^{2-} composites obtained at an initial SO_4^{2-} concentration of 1000 mg/L.

Table S1. Kinetic parameters for the sorption of Cu^{2+} onto the XG-based cryogels.

Table S2. Atomic percentages (%) of elements present on the surface of the SN and DN cryogels after interaction with SO_4^{2-} (C_i = 500 mg/L).

Table S3. Atomic percentages (%) of elements present on the surface of the SN and DN cryogels after interaction with SO_4^{2-} (C_i = 1000 mg/L).

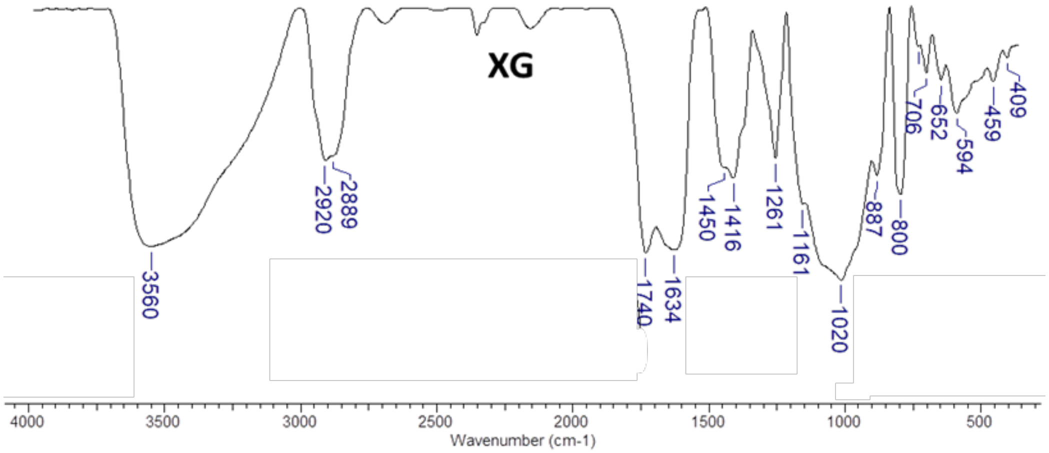

Figure S1. FTIR spectrum of uncross-linked XG.

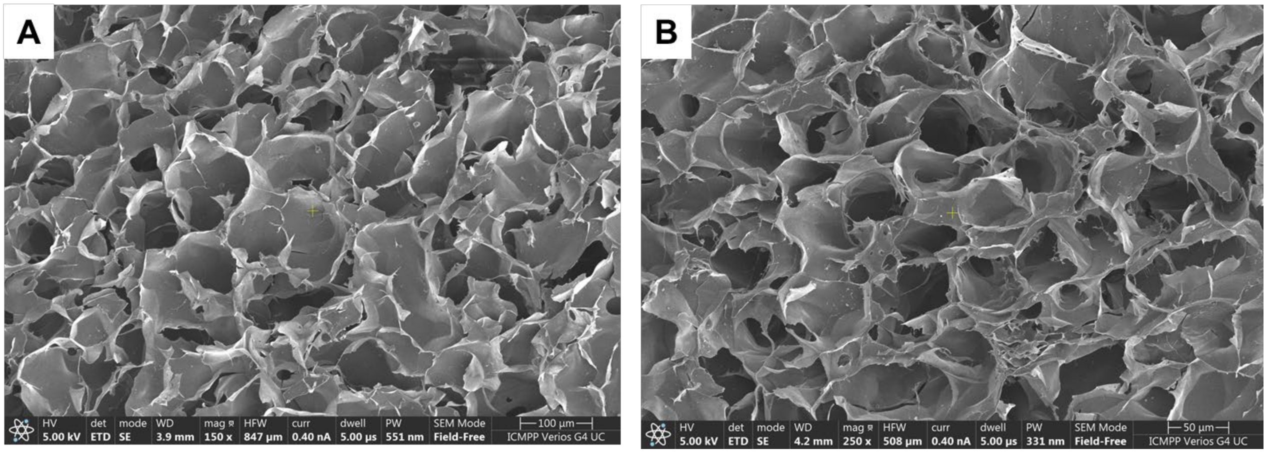

Figure S2. SEM micrographs of the SN.X2 cryogels at different magnifications: (A) 150x; (B) 250x.

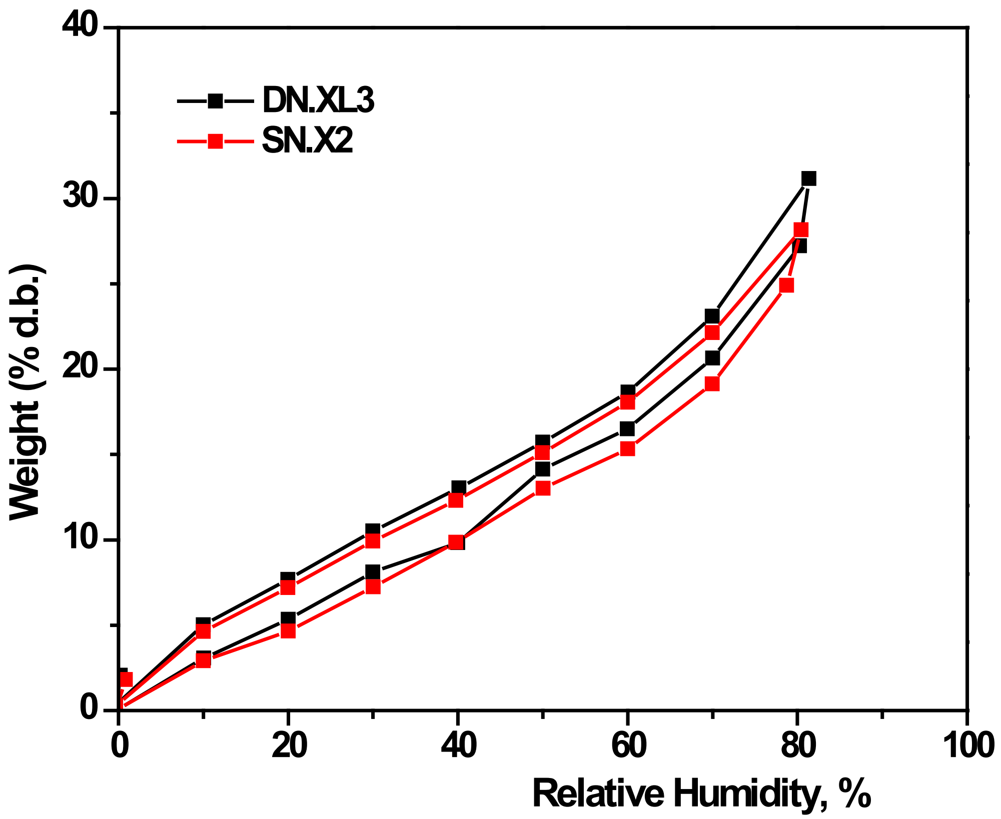

Figure S3. Sorption-desorption isotherms of the XG-based cryogels.

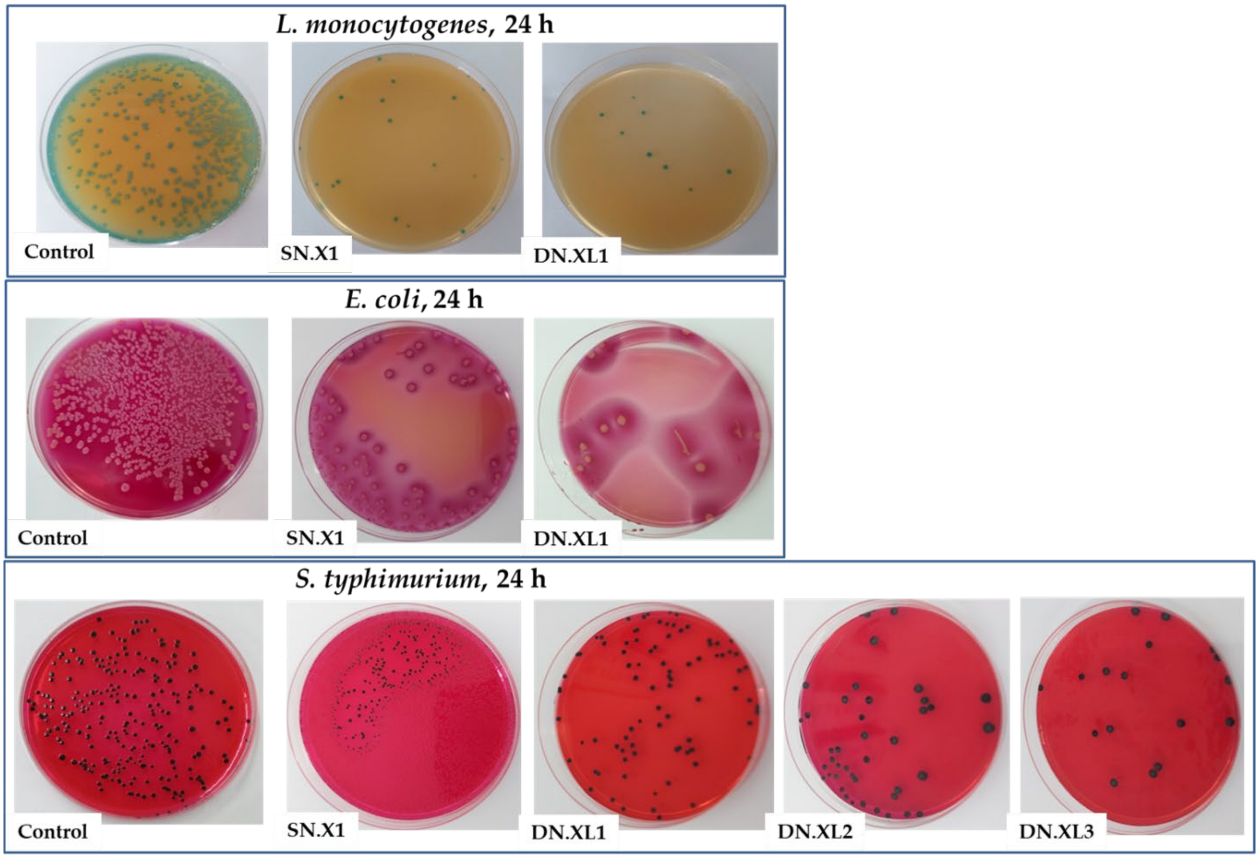

Figure S4. Representative photographs showing the antimicrobial effects of the tested samples against *Listeria monocytogenes*, *Escherichia coli*, and *Salmonella Typhimurium* after 24 h of incubation. For each organism, the control represents bacterial growth in the absence of the tested sample, while SN.X2, DN.XL1, DN.XL2, and DN.XL3 represent the corresponding treatments. The reduction in the number of visible colonies relative to the control indicates inhibition of bacterial growth. The figure illustrates the differential susceptibility of the three bacterial species to the tested samples, with progressively fewer colonies generally observed in treated plates compared with their respective controls.

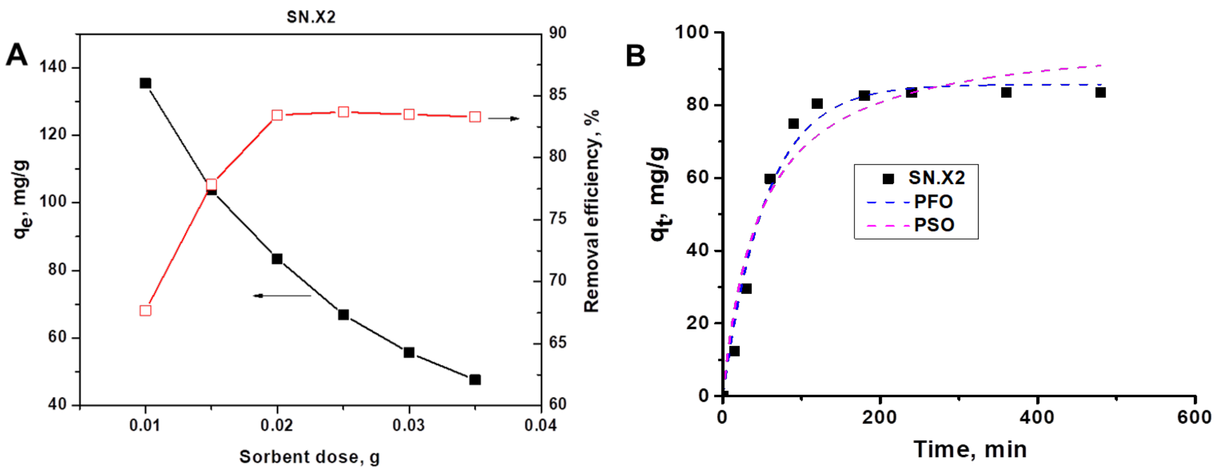

Figure S5. (A) Influence of sorbent dose on the sorption capacity (q_e, mg/g) and removal efficiency (RE,%) of Cu^{2+} ions for SN.X2 cryogels; (B) Influence of contact time on the sorption capacity of Cu^{2+} ions for SN.X2 cryogels.

The kinetic data were quantitatively analyzed by fitting two models: the pseudo-first-order (PFO) model (Eq. S1), pseudo-second-order the PSO model (Eq. S2):

$$q_t = q_e(1 - e^{-k_1 t}) \quad (Eq. S1)$$

$$q_t = \frac{k_2 q_e^2 t}{1 + k_2 q_e t} \quad (Eq. S2)$$

where: q_e and q_t - the amount of Cu^{2+} sorbed at equilibrium (mg/g) and a time t, respectively, k_1 - the rate constant of the PFO kinetic model (min^{-1}), k_2 the rate constant of PSO kinetic model (g/mg × min).

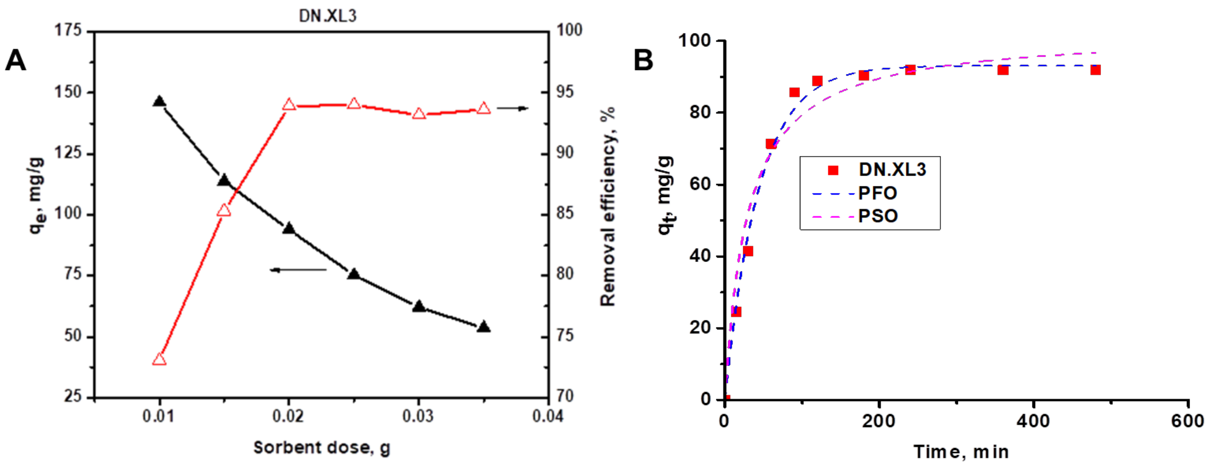

Figure S6. (A) Influence of sorbent dose on the sorption capacity (q_e, mg/g) and removal efficiency (RE,%) of Cu^{2+} ions for DN.XL3 cryogels; (B) Influence of contact time on the sorption capacity of Cu^{2+} ions for DN.XL3 cryogels.

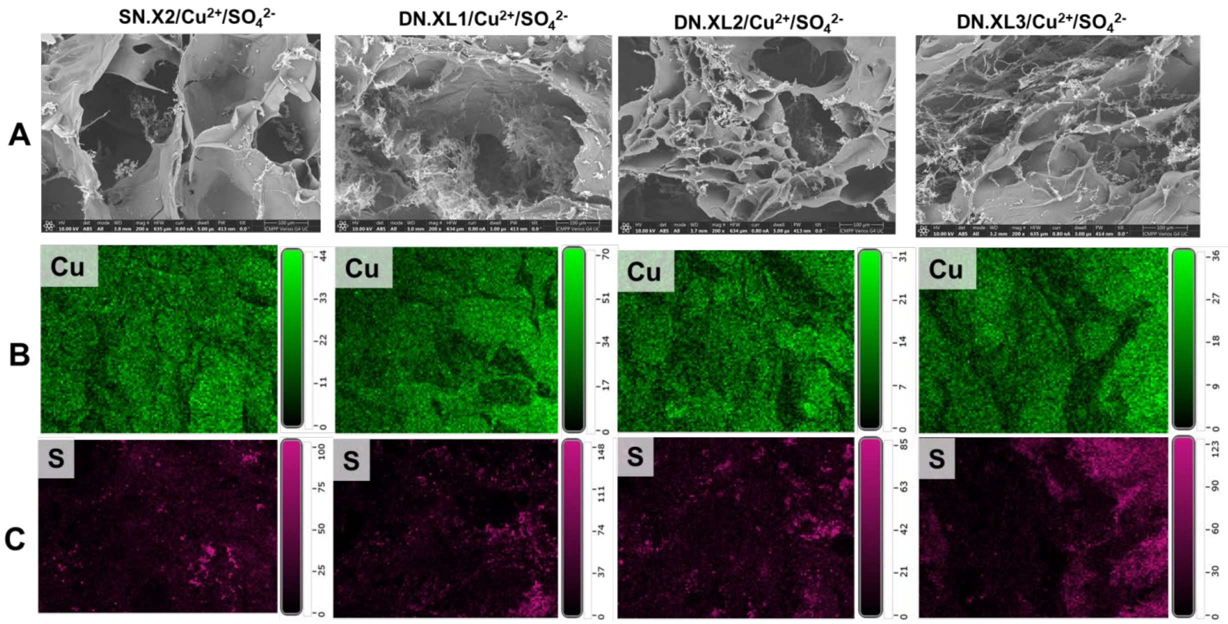

Figure S7. (A) SEM micrographs of SN/Cu^{2+}/ SO_4^{2-} and DN/ Cu^{2+}/ SO_4^{2-} composites obtained at an initial SO_4^{2-} concentration of 1000 mg/L; (B) EDX mapping of SN/Cu^{2+}/ SO_4^{2-} and DN/ Cu^{2+}/ SO_4^{2-} composites obtained at an initial SO_4^{2-} concentration of 1000 mg/L showing the presence of Cu on the surface of composites; (C) EDX mapping of SN/Cu^{2+}/ SO_4^{2-} and DN/ Cu^{2+}/ SO_4^{2-} composites obtained at an initial SO_4^{2-} concentration of 1000 mg/L showing the presence of S on the surface of composites.

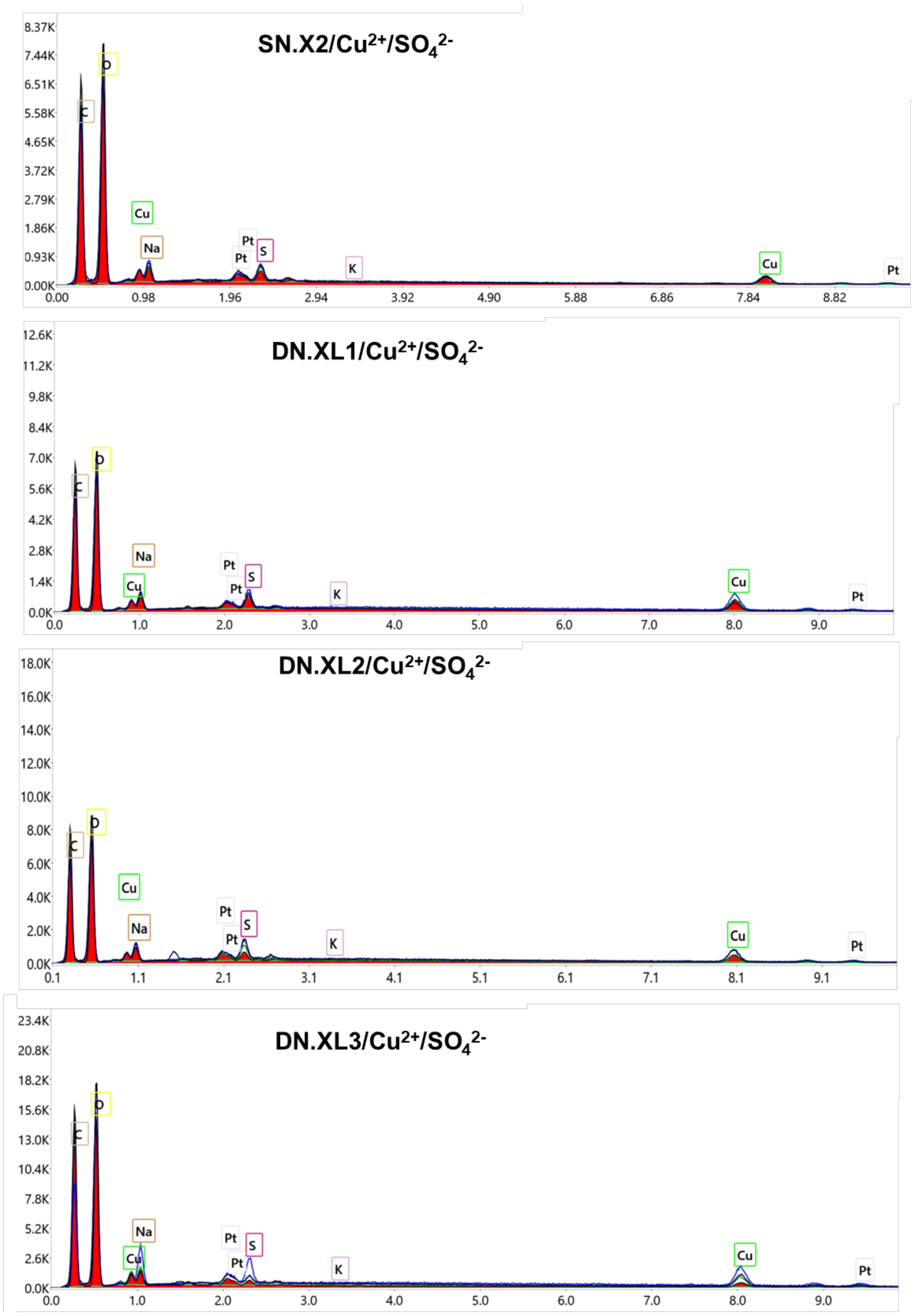

Figure S8. EDX profiles of SN/Cu^{2+}/ SO_4^{2-} and DN/ Cu^{2+}/ SO_4^{2-} composites obtained at an initial SO_4^{2-} concentration of 500 mg/L.

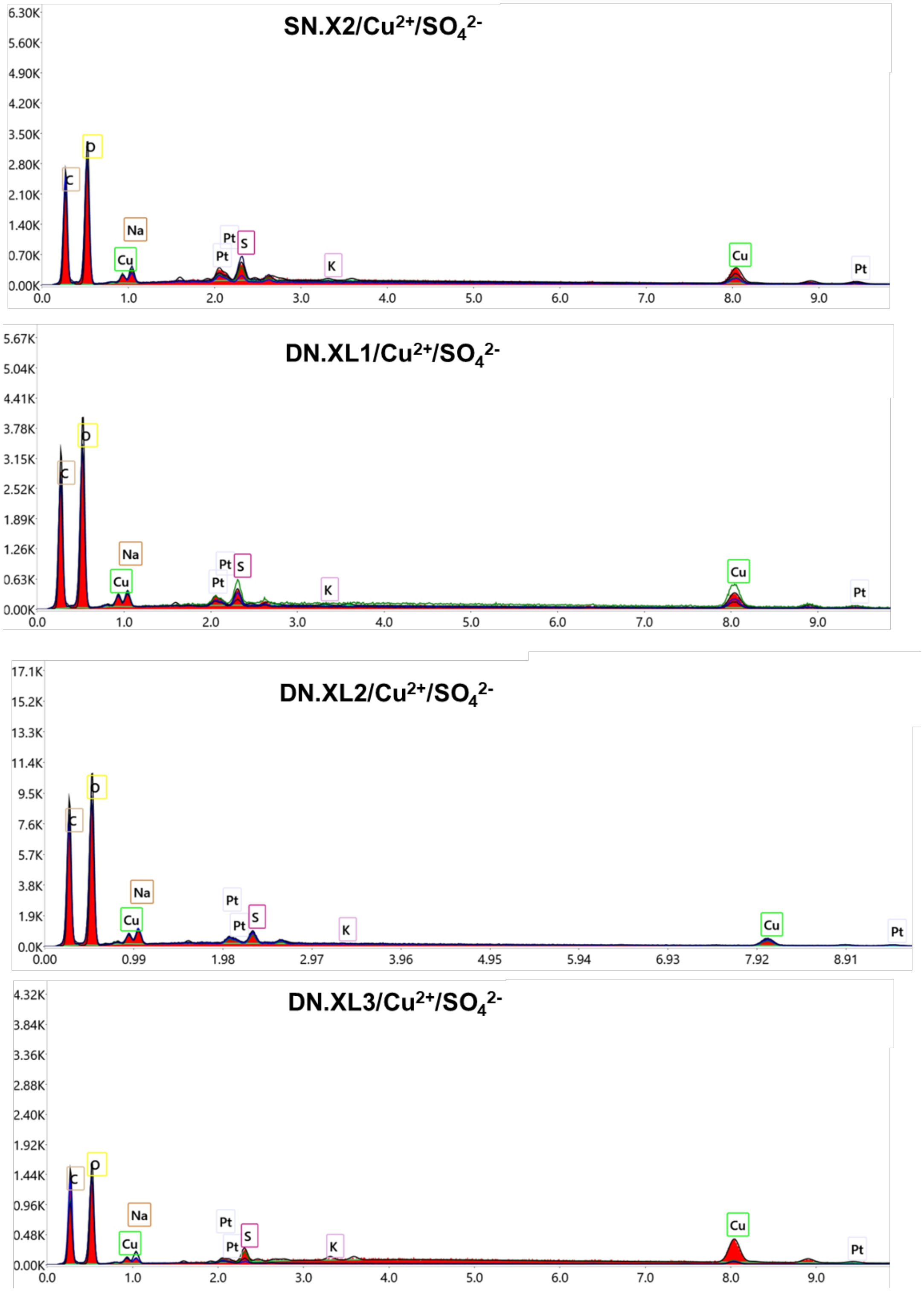

Figure S9. EDX profiles of SN/Cu^{2+}/ SO_4^{2-} and DN/ Cu^{2+}/ SO_4^{2-} composites obtained at an initial SO_4^{2-} concentration of 1000 mg/L.

Table S1. Kinetic parameters for the sorption of Cu^{2+} onto the XG-based cryogels.

**Kinetic model**	**Kinetic parameters**	**SN.X2**	**DN.XL3**
	$q_{m,\ exp}$ (mg/g)	83.45	91.92
**PFO**	$q_{m,\ calc.}$ (mg/g)	85.71	93.13
	k_1, min^{-1}	0.018	0.023
	R^2	0.979	0.993
	χ^2	22.49	7.54
**PSO**	$q_{m,\ calc.}$ (mg/g)	99.81	102.61
	k_2, (g/mg x min)	2.124 x 10^{-4}	3.341 x 10^{-4}
	R^2	0.942	0.97
	χ^2	61.66	9.95

Table S2. Atomic percentages (%) of elements present on the surface of the SN and DN cryogels after interaction with SO_4^{2-} (C_i = 500 mg/L).

**Sample/Element**	**SN.X2**	**DN.XL1**	**DN.XL2**	**DN.XL3**
**C**	48.10 ± 0.42	49.17 ± 0.92	49.53 ± 0.41	49,47 ± 0.44
**O**	48.08 ± 0.54	45.67 ± 0.74	44.80 ± 0.78	44.57 ± 0.25
**Na**	1.67 ± 0.25	1.50 ± 0.22	2.09 ± 0.34	1.30 ± 0.33
**S**	0.58 ± 0.13	0.61 ± 0.08	1.33 ± 0.17	2.08 ± 0.15
**K**	0.08 ± 0.04	0.07 ± 0.04	0.07 ± 0.05	0.08 ± 0.04
**Cu**	1.03 ± 0.11	1.33 ± 0.46	1.90 ± 0.45	2.30 ± 0.43
**Pt**	0.28 ± 0.04	0.37 ± 0.13	0.28 ± 0.15	0.20 ± 0.07

Table S3. Atomic percentages (%) of elements present on the surface of the SN and DN cryogels after interaction with SO_4^{2-} (C_i = 1000 mg/L).

**Sample/Element**	**SN.X2**	**DN.XL1**	**DN.XL2**	**DN.XL3**
**C**	47.30 ± 1.51	48.63 ± 0.47	48.10 ± 0.83	47.18 ± 0.64
**O**	48.18 ± 1.10	46.18 ± 0.57	46.03 ± 1.59	45.70 ± 1.42
**Na**	1.15 ± 0.95	1.13 ± 0.19	1.00 ± 0.14	1.00 ± 0.35
**S**	1.91 ± 0.26	2.40 ± 0.07	2.63 ± 0.17	2.83 ± 0.26
**K**	0.06 ± 0.12	0.03 ± 0.04	0.08 ± 0.04	0.07 ± 0.05
**Cu**	1.17 ± 0.17	1.40 ± 0.19	1.93 ± 0.45	2.13 ± 0.29
**Pt**	0.23 ± 0.11	0.23 ± 0.05	0.23 ± 0.18	0.20 ± 0.12
